# The optimal vaccination strategy to control COVID-19: a modeling study in Wuhan City, China

**DOI:** 10.1186/s40249-021-00922-4

**Published:** 2021-12-28

**Authors:** Ze-yu Zhao, Yan Niu, Li Luo, Qing-qing Hu, Tian-long Yang, Mei-jie Chu, Qiu-ping Chen, Zhao Lei, Jia Rui, Cheng-long Song, Sheng-nan Lin, Yao Wang, Jing-wen Xu, Yuan-zhao Zhu, Xing-chun Liu, Meng Yang, Jie-feng Huang, Wei-kang Liu, Bin Deng, Chan Liu, Zhuo-yang Li, Pei-hua Li, Yan-hua Su, Ben-hua Zhao, Wen-long Huang, Roger Frutos, Tian-mu Chen

**Affiliations:** 1 State Key Laboratory of Molecular Vaccinology and Molecular Diagnostics, School of Public Health, Xiamen University, 4221-117 South Xiang’an Road, Xiang’an District, Xiamen, 361102 Fujian People’s Republic of China; 2 Cirad, UMR 17, Intertryp, Université de Montpellier, Montpellier, France; 3 Chinese Center for Disease Control and Prevention, Beijing, People’s Republic of China; 4 Division of Public Health, School of Medicine, University of Utah, Utah, USA; 5 Medical Insurance Office, Xiang’an Hospital of Xiamen University, Xiamen, Fujian People’s Republic of China; 6 Department of Data Science, College of Natural Sciences, Colorado State University, Fort Collins, CO USA; 7 Fujian Provincial Center for Disease Control and Prevention, 76 Jintai Road, Gulou District, Fuzhou, Fujian People’s Republic of China

**Keywords:** SARS-CoV-2, Transmissibility, Age-specific model, Vaccination strategy, Effectiveness

## Abstract

**Background:**

Reaching optimal vaccination rates is an essential public health strategy to control the coronavirus disease 2019 (COVID-19) pandemic. This study aimed to simulate the optimal vaccination strategy to control the disease by developing an age-specific model based on the current transmission patterns of COVID-19 in Wuhan City, China.

**Methods:**

We collected two indicators of COVID-19, including illness onset data and age of confirmed case in Wuhan City, from December 2, 2019, to March 16, 2020. The reported cases were divided into four age groups: group 1, ≤ 14 years old; group 2, 15 to 44 years old; group 3, 44 to 64 years old; and group 4, ≥ 65 years old. An age-specific susceptible-exposed-symptomatic-asymptomatic-recovered/removed model was developed to estimate the transmissibility and simulate the optimal vaccination strategy. The effective reproduction number (*R*_eff_) was used to estimate the transmission interaction in different age groups.

**Results:**

A total of 47 722 new cases were reported in Wuhan City from December 2, 2019, to March 16, 2020. Before the travel ban of Wuhan City, the highest transmissibility was observed among age group 2 (*R*_eff_ = 4.28), followed by group 2 to 3 (*R*_eff_ = 2.61), and group 2 to 4 (*R*_eff_ = 1.69). China should vaccinate at least 85% of the total population to interrupt transmission. The priority for controlling transmission should be to vaccinate 5% to 8% of individuals in age group 2 per day (ultimately vaccinated 90% of age group 2), followed by 10% of age group 3 per day (ultimately vaccinated 90% age group 3). However, the optimal vaccination strategy for reducing the disease severity identified individuals ≥ 65 years old as a priority group, followed by those 45–64 years old.

**Conclusions:**

Approximately 85% of the total population (nearly 1.2 billion people) should be vaccinated to build an immune barrier in China to safely consider removing border restrictions. Based on these results, we concluded that 90% of adults aged 15–64 years should first be vaccinated to prevent transmission in China.

**Graphical Abstract:**

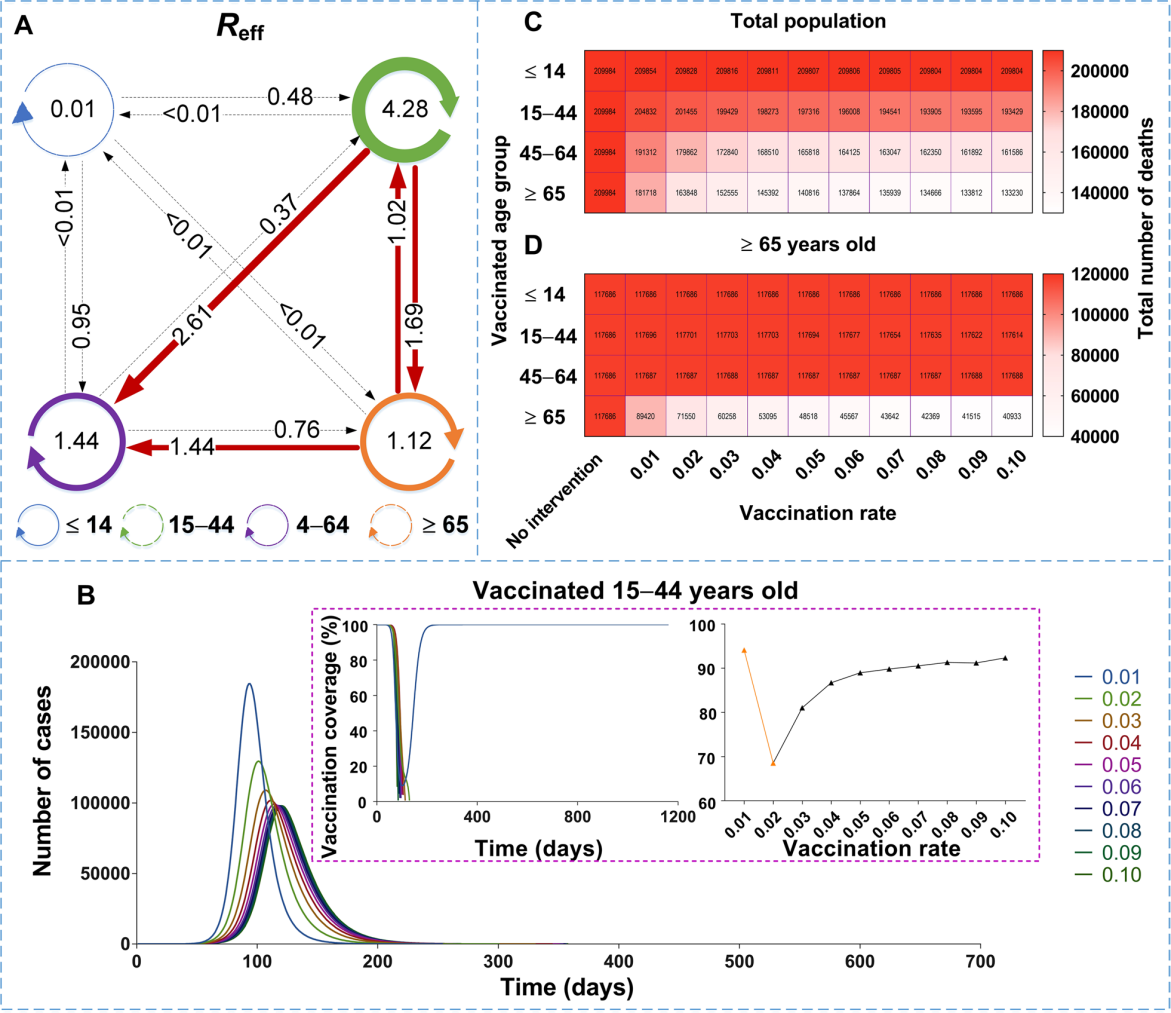

**Supplementary Information:**

The online version contains supplementary material available at 10.1186/s40249-021-00922-4.

## Background

Currently, the coronavirus disease 2019 (COVID-19) pandemic has become a heavy burden worldwide. As of November 15, 2021, 253 163 330 confirmed cases have been reported by the World Health Organization [1]. Several effective non-pharmaceutical interventions (NPIs) such as travel bans, social distancing, case isolation, and mask-wearing have been applied in China to mitigate the epidemic [2]. However, the implementation of NPIs has varied considerably across countries [3]. Therefore, vaccine and antiviral therapies are essential to prevent the spread of COVID-19 and control the epidemic [4]. Recently, studies have shown that the vaccine efficacy (VE) ranged from 90 to 94% (with two doses of vaccination) for controlling infection of the ancestral virus [5–7], and even 67% to 88% (with two doses) for the Delta variant [8]. Furthermore, the vaccine’s effectiveness for controlling disease severity and death was higher than that for reducing infection, especially with the great heterogeneity in different age groups [9]. Therefore, optimal vaccination protocols for different age groups should be simulated to help guide an appropriate vaccine strategy.

Most current studies used dynamic models of severe acute respiratory syndrome coronavirus 2 (SARS-CoV-2), such as the susceptible-exposed-infectious-removed (SEIR) model to clarify early transmission, and the multi-host model to estimate transmissibility [10, 11]. Moreover, our previous study adopted an age-specific susceptible-exposed-symptomatic-asymptomatic-recovered/removed (SEIAR) model to estimate the relative transmissibility in different age groups, but did not simulate the vaccine effects [12]. Furthermore, the other study adopted a SEIR model to access the vaccine effects in different populations suggested that a VE ≥ 50% would be sufficient to mitigate the pandemic [13]. Some modeling studies recommended targeting older age groups as an optimal strategy for controlling death [14, 15], which was suitable for reducing the disease severity and death rates in some Western countries. Furthermore, some studies have simulated vaccine effectiveness based on the contact matrix and assumption of basic reproduction number (*R*_0_) [13, 15, 16], but did not analyze the transmission pattern in different age groups through first-hand data. In this study, we employed an age-specific SEIAR model to explore the transmission features, compare the transmissibility and assess the vaccine effectiveness in different age groups.

## Methods

### Data collection and study design

**Fig. 1:**
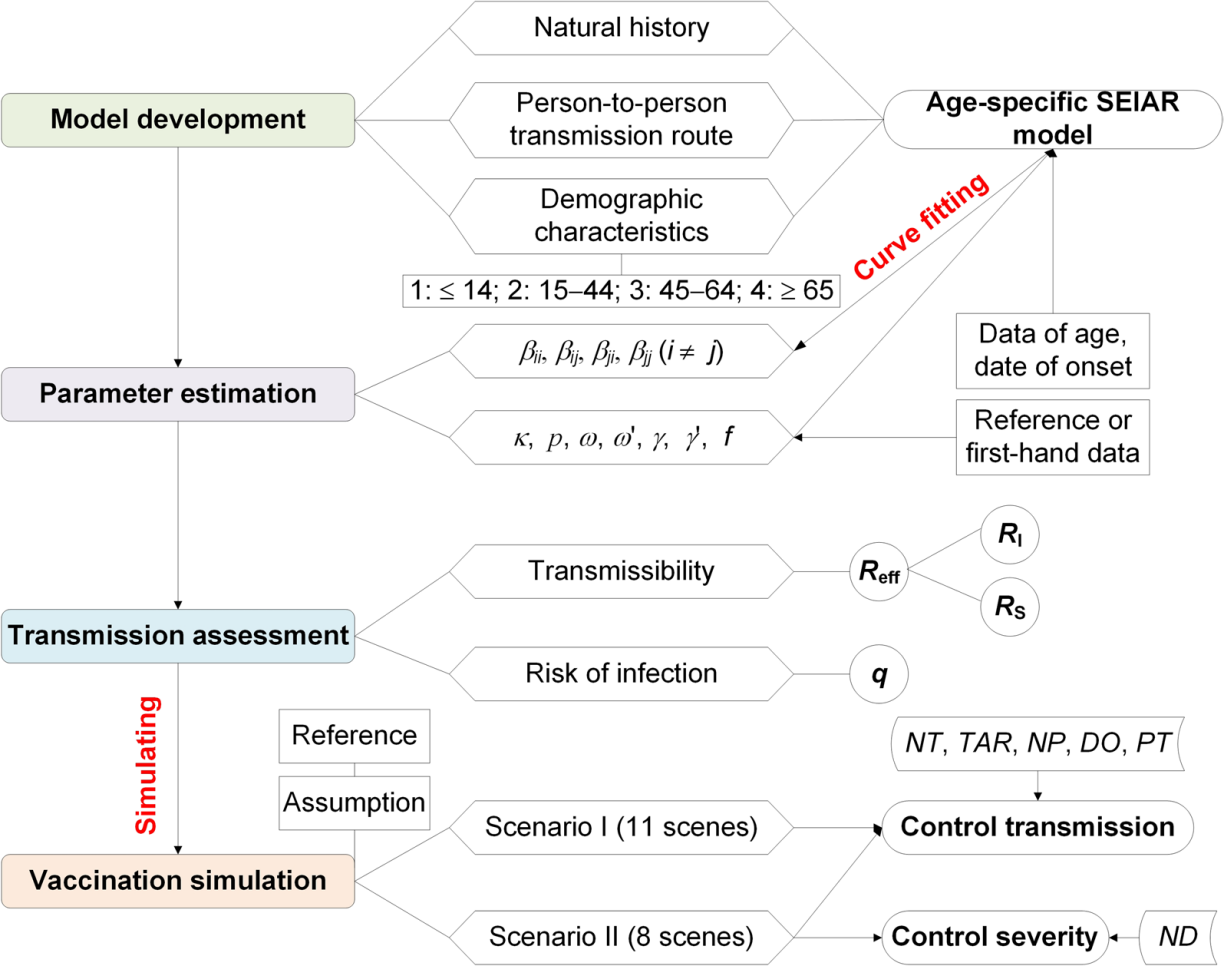
The flowchart of the study design

Data including age and date of onset of COVID-19 in Wuhan City from December 2, 2019, to March 16, 2020, were collected from a previous study [17] (Additional file 1: Table S1). Our study included four phases including model development, parameter estimation, transmission assessment, and vaccination simulation (Fig. 1). The subscripts *i* and *j* (*i* ≠ *j*) are referred to as age groups 1 to 4, respectively. Age group 1 was defined as ≤ 14 years old; group 2, 15–44 years old; group 3, 45–64 years old; and group 4, ≥ 65 years old.

### Model development

We adopted an age-specific SEIAR model to estimate the transmissibility (Model 1), and two vaccinated age-specific SEIAR models (Model 2 and Model 3) to simulate the vaccination effects.

According to our previous study, we developed a multi-host model and age-specific model and further applied the age-specific SEIAR model to estimate the disease transmission [11, 12]. In the model, the total population (*N*) was divided into five categories, susceptible (*S*), exposed (*E*), symptomatic (*I*), asymptomatic (*A*), and recovered/removed (*R*). Table 1 presents the definitions in detail.

**Table 1:** Variables in the age-specific model

Variables	Description	Unit
S	Susceptible individuals	Individuals
E	Exposed individuals	Individuals
I	Infectious individuals	Individuals
A	Asymptomatic individuals	Individuals
R	Recovered/Removed individuals	Individuals
V_1_	Vaccinated individuals without immunity	Individuals
V_2_	Vaccinated individuals with immunity	Individuals
N	Total number of population	Individuals

The model conditions or assumptions were as follows:

- Susceptible individuals infected by contact with symptomatic or asymptomatic patients. SARS-CoV-2 can be transmitted within a given age group *i* with a relative transmission rate (*β*_*ii*_) and between age groups *i* and *j* with a relative transmission rate (*β*_*ij*_).
- The proportion of asymptomatic infections was defined as *p*. The exposed individuals would become symptomatic and asymptomatic after an incubation period (1/*ω*) and a latent period (1/*ω*’). In the model, the incubation period was assumed to be equal to the latent period.
- The transmissibility of the virus from asymptomatic and symptomatic patients differs by factor *κ*.
- The symptomatic and asymptomatic patients are converted to recovered/removed persons after an infectious period of 1/*γ* and 1/*γ*’, respectively.

**Fig. 2:**
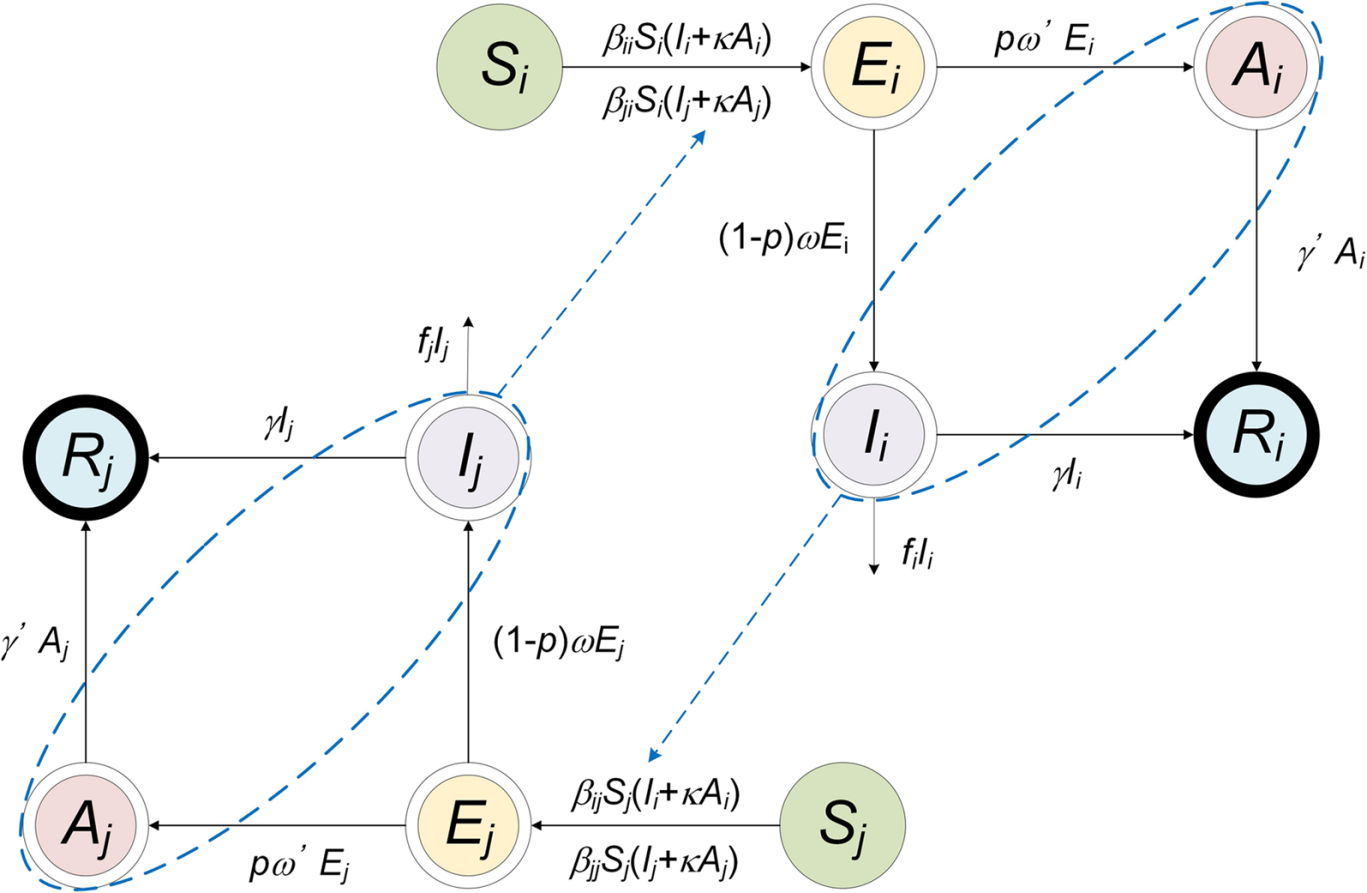
The flowchart of the age-specific SEIAR model (Model 1)

A flowchart of Model 1 is presented in Fig. 2. The equations of the model are as follows:

$$i\ne j$$

$$\frac{d{S}_{i}}{dt}=-{\beta }_{ii}{S}_{i}\left({I}_{i}+\kappa {A}_{i}\right)-{\beta }_{ji}{S}_{i}\left({I}_{j}+\kappa {A}_{j}\right)-{\delta }_{i}{S}_{i}$$

$$\frac{d{E}_{i}}{dt}={\beta }_{ii}{(V}_{1i}+({1-\lambda ){V}_{2i}+S}_{i})\left({I}_{i}+\kappa {A}_{i}\right)+{\beta }_{ji}{(V}_{1i}+({1-\lambda ){V}_{2i}+S}_{i})\left({I}_{j}+\kappa {A}_{j}\right)-\left(1-p\right)\omega {E}_{i}-p{\omega }^{^{\prime}}{E}_{i}$$

$$\frac{d{I}_{i}}{dt}=\left(1-p\right)\omega {E}_{i}-\gamma {I}_{i}-{f}_{i}{I}_{i}$$

$$\frac{d{A}_{i}}{dt}=p{\omega }^{^{\prime}}{E}_{i}-{\gamma }^{^{\prime}}{A}_{i}$$

$$\frac{d{R}_{i}}{dt}={\gamma I}_{i}+{{\gamma }^{^{\prime}}A}_{i}$$

$$\frac{d{V}_{1i}}{dt}={\delta }_{i}{S}_{i}-{\beta }_{ii}{V}_{1i}\left({I}_{i}+\kappa {A}_{i}\right)-{\beta }_{ji}{V}_{1i}\left({I}_{j}+\kappa {A}_{j}\right)-\varphi {V}_{1i}$$

$$\frac{d{V}_{2i}}{dt}=\varphi {V}_{1i}-{\left(1-\lambda \right)\beta }_{ii}{V}_{2i}\left({I}_{i}+\kappa {A}_{i}\right)-{(1-\lambda )\beta }_{ji}{V}_{2i}\left({I}_{j}+\kappa {A}_{j}\right)$$

$$\frac{d{S}_{j}}{dt}=-{\beta }_{jj}{S}_{j}\left({I}_{j}+\kappa {A}_{j}\right)-{\beta }_{ji}{S}_{j}\left({I}_{i}+\kappa {A}_{i}\right)-\delta {S}_{j}$$

$$\frac{d{E}_{j}}{dt}={\beta }_{jj}{(V}_{1j}+({1-\lambda ){V}_{2j}+S}_{j})\left({I}_{j}+\kappa {A}_{j}\right)+{\beta }_{ji}{(V}_{1j}+({1-\lambda ){V}_{2j}+S}_{j})\left({I}_{i}+\kappa {A}_{i}\right)-\left(1-p\right)\omega {E}_{j}-p{\omega }^{^{\prime}}{E}_{j}$$

$$\frac{d{I}_{j}}{dt}=\left(1-p\right)\omega {E}_{j}-\gamma {I}_{j}-{f}_{i}{I}_{j}$$

$$\frac{d{A}_{j}}{dt}=p{\omega }^{^{\prime}}{E}_{j}-{\gamma }^{^{\prime}}{A}_{j}$$

$$\frac{d{R}_{j}}{dt}={\gamma I}_{j}+{{\gamma }^{^{\prime}}A}_{j}$$

$$\frac{d{V}_{1j}}{dt}={\delta }_{j}{S}_{j}-{\beta }_{jj}{V}_{1j}\left({I}_{j}+\kappa {A}_{j}\right)-{\beta }_{ij}{V}_{1j}\left({I}_{i}+\kappa {A}_{i}\right)-\varphi {V}_{1j}$$

$$\frac{d{V}_{2j}}{dt}=\varphi {V}_{1j}-{\left(1-\lambda \right)\beta }_{jj}{V}_{2j}\left({I}_{j}+\kappa {A}_{j}\right)-{(1-\lambda ){\beta }_{ij}V}_{2j}\left({I}_{i}+\kappa {A}_{i}\right)$$

$$N={S}_{i}+{E}_{i}+{I}_{i}+{A}_{i}+{R}_{i}+{V}_{1i}+{V}_{2i}$$

The left side of the differential equation shows the instantaneous change rates of *S*, *E*, *I*, *A*, and *R,* at time *t*. The subscripts *i* and *j* (*i* ≠ *j*) represent age groups 1–4.

**Fig. 3:**
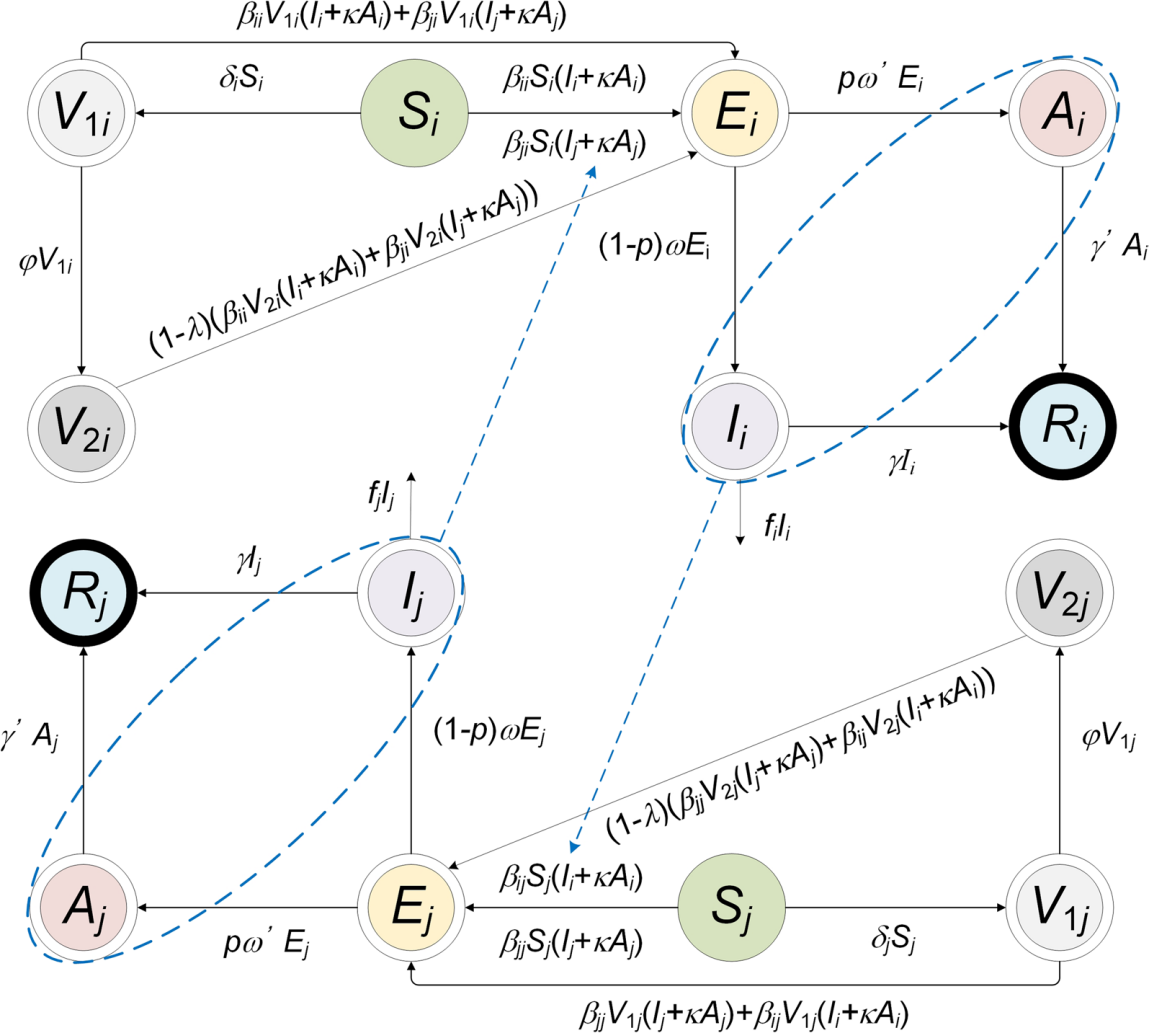
The flowchart of vaccinated age-specific SEIAR model (Model 2). *i* and *j* represent age ≤ 14, 15‒44, 45‒64, and ≥ 65, respectively (*i* ≠ *j*)

In the age-specific SEIAR model, two compartments were added, which were defined as vaccinated individuals without immunity (*V*_*1*_) and vaccinated individuals with immunity (*V*_*2*_). We built two models (Model 2 and Model 3) based on the following assumptions:

A flowchart of Model 2 is shown in Fig. 3. The following assumption should be added to the age-specific SEIAR model:

- Assuming that only susceptible individuals were vaccinated, *δ* is considered the daily vaccination rate.
- Vaccinated individuals without immunity, infected by contact with symptomatic or asymptomatic cases; the relative transmission rate is also *β*_*ii*_ within the age group and *β*_*ij*_ between age groups.
- Vaccinated individuals without immunity will become immune after a period. Protective antibodies will occur within 1/*φ* days, which is described as the period in which vaccinated individuals without immunity will become immune.
- The COVID-19 vaccine has a VE defined as *λ*. Vaccinated individuals with immunity are infected by contact with symptomatic or asymptomatic patients. The infection rate is defined as 1–*λ*.

The equations used in the model (Model 2) are in Additional file 2: Text S1.

We assumed that the VE of COVID-19 was similar to what was previously reported for the H1N1 pandemic [18] (Model 3), which quantified the different protective effects as follows: VE_*S*_ refers to VE against susceptibility, VE_*I*_ as VE against infectiousness, and VE_*P*_ as VE against pathogenicity or symptomatic illness has given infection. In addition, according to the theory of herd immunity [19], we have considered the coefficient of herd immunity (*θ*), defined as the proportion of herd immunity, which was calculated as follows:

$$\theta =1-\frac{Total~number~of~new~cases}{N}$$

Here, we added three parameters into the model, including *x* referring to a decreased proportion of VE against susceptibility, *y* referring to as a decreasing proportion of VE against infectivity, and *z* referring to as a decreasing proportion of VE against pathogenicity. The equations used for calculation are as follows:

$$x=1-{VE}_{S}$$

$$y=1-{VE}_{I}$$

$$z=1-{VE}_{p}$$

**Fig. 4:**
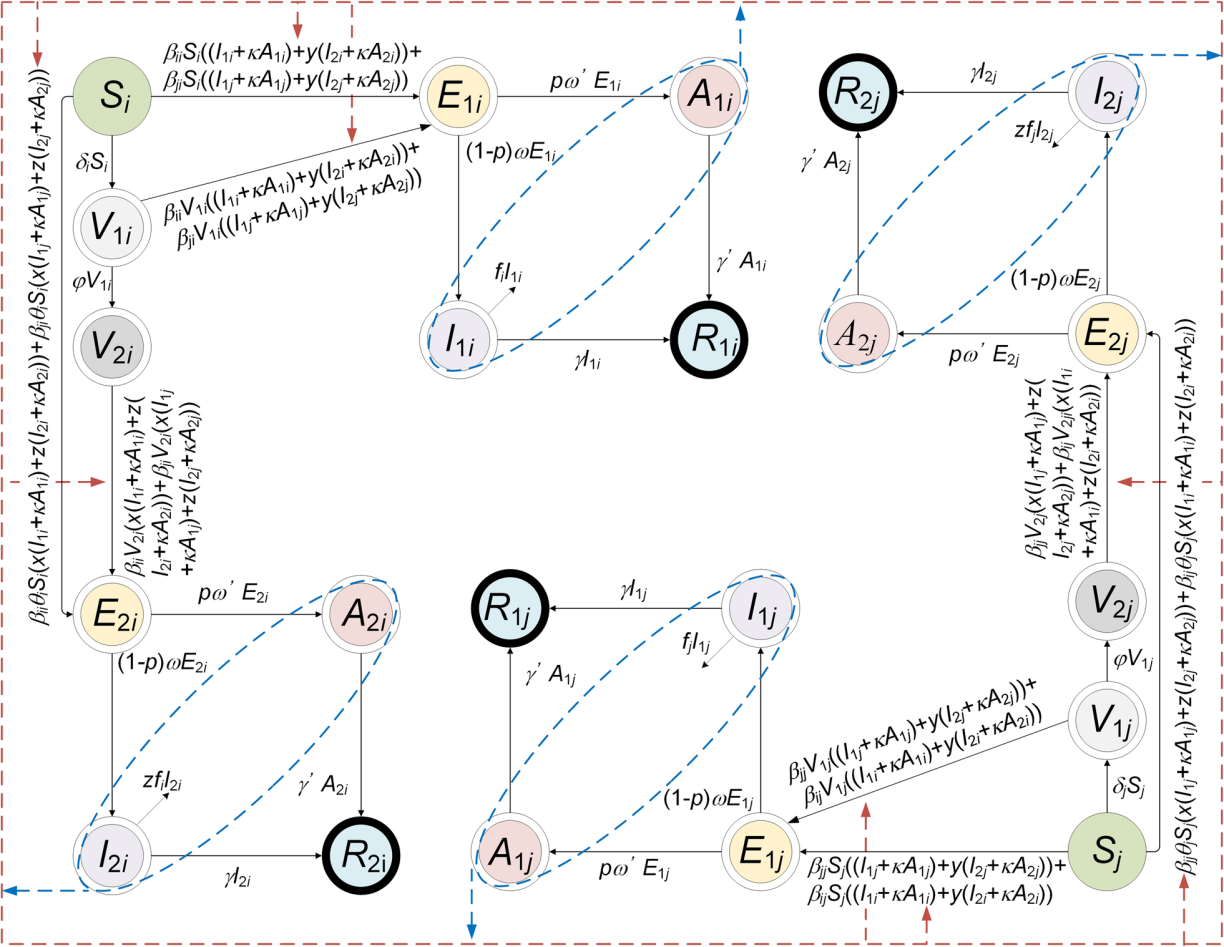
The flowchart of vaccinated age-specific SEIAR model (Model 3, considering the vaccine efficacy of susceptibility and infectivity). *i* and *j* represent age ≤ 14, 15‒44, 45‒64, and ≥ 65, respectively (*i* ≠ *j*)

A flowchart of Model 3 is presented in Fig. 4. The following assumption should be added to the age-specific SEIAR model:

- Once herd immunity has been attained, susceptible individuals are infected following contact with symptomatic or asymptomatic patients. The proportion of herd immunity is *θ*.
- Vaccinated individuals without immunity (*V*_1_) would be infected by contact with two types of cases, namely, non-vaccinated symptomatic/asymptomatic cases and vaccinated symptomatic/asymptomatic cases. Meanwhile, the infectivity of *I*_2_ and *A*_2_ would decrease by a proportion of *y*.
- Vaccinated individuals with immunity (*V*_2_) would be infected following contact with two types of cases, namely, non-vaccinated symptomatic/asymptomatic cases and vaccinated symptomatic/asymptomatic cases. The susceptibility of *V*_2_ would be decreased by a proportion of *x* and the pathogenicity of SARS-CoV-2 would decrease by a proportion of *z*.

The equations used in the model (Model 3) are Additional file 2: Text S1.

### Vaccination simulation scenarios

**Fig. 5:**
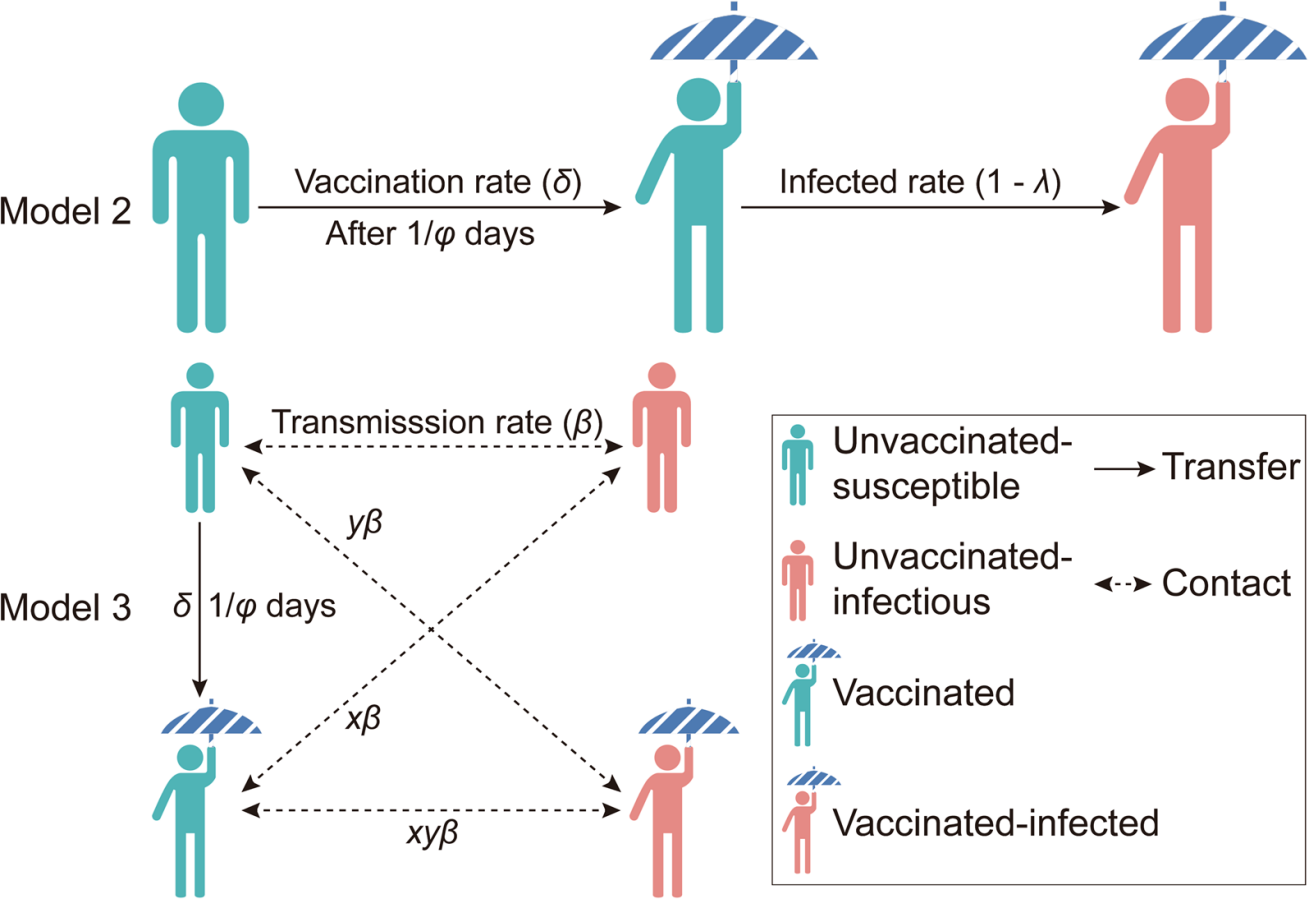
The process of vaccination in Model 2 and Model 3. Parameter *δ*, *λ*, *β*, *φ*, *x* and *y* represent vaccination rate, vaccine efficacy, transmission rate, immune relative rate, decreasing proportion of vaccine efficacy against susceptibility, and decreasing proportion of vaccine efficacy against infectivity, respectively

In this study, we simulated the vaccine’s effects based on the stage before travel ban in Wuhan City (stage 1). We developed two scenarios (referred to as scenario I and scenario II) to assess the vaccine’s effects (Fig. 5). Thereafter, we built 19 sub-scenarios (defined as scenes) based on the above two scenarios. The conditions of the scenarios were as follows:

- Scenario I: According to Model 2, we assumed that susceptible people could be vaccinated at a rate of *δ* and achieve immunity after 1/*φ* days. Previously immune individuals could be infected at a rate of 1−*λ*. We assumed the VE of COVID-19 is similar to that of measles and influenza vaccines and simulates effects by changing the parameters *δ,* *φ,* and *λ*. The conditions of the 11 scenes (I to XI) are shown in Additional file 3: Table S2.
- Scenario II: Model 3 also assumes that susceptible people could be vaccinated at a rate of *δ* and achieve immunity after 1/*φ* days. Furthermore, we assumed that if immune people become ill, infectivity could be reduced after vaccination; the remaining ratio after reduction is *y*. Susceptibility could be reduced after vaccination if immune people contacted disease cases, and the remaining ratio after reduction was *x*. If contact occurred between immune people and cases, both parameters could be reduced. The future vaccine has similar effects as influenza A (H1N1) and simulated vaccine effects by changing the parameters *δ*, x, y, and *z*. The total population was divided into two groups, vaccinated and non-vaccinated. The conditions of the eight scenes (XII to XIX) are shown in Additional file 3: Table S2.

Additionally, we assumed that the severity of disease would be decreased after vaccination, and the coefficient was the same as the parameter *z* = 0.33 (decreasing the proportion of VE) in scenes XIV–XVII of scenario II.

### Parameter estimation

In this study, several parameters were adopted to develop the model, and the description, value, and method were listed in Table 2. The parameter *κ* refers to the relative transmissibility rate of asymptomatic to symptomatic individuals. In the model, *κ* is set to 0.65. The most important reason is that a study reported that 4.11% of individuals would become infected after close contact with asymptomatic patients, versus 6.3% for individuals infected after close contact with symptomatic patients [20]. In addition, it has been reported that the transmissibility of symptomatic patients is 3.9 times that of asymptomatic patients, and an asymptomatic individual may contribute to 11 infectious cases [21, 22]. In their dynamic model, the parameter *κ* was set to 1.0 [12].

**Table 2:** Description and source of parameters in the age-specific model

Parameter	Description	Unit	Value	Range	Method
*β*_ii_^*^	Transmission relative rate among age group *i*	Individuals^-1^·days^-1^	–	≥ 0	Curve fitting
*β*_ij_^*^	Transmission relative rate from age group *i* to *j*	Individuals^-1^·days^-1^	–	≥ 0	Curve fitting
*β*_ji_^*^	Transmission relative rate from age group *j* to *i*	Individuals^-1^·days^-1^	–	≥ 0	Curve fitting
*β*_jj_^*^	Transmission relative rate among age group *i*	Individuals^-1^·days^-1^	–	≥ 0	Curve fitting
*κ*	Relative transmissibility rate of asymptomatic to symptomatic individuals	1	0.65	0–1	[12, 21, 22]
*p*	Proportion of the asymptomatic	1	0.36	0.016–0.78	[23, 24]
*ω*	Incubation relative rate	Days^-1^	0.2	0.05556–0.5	[20, 25–27]
*ω*'	Latent relative rate	Days^-1^	0.2	0.05556–0.5	[20, 25–27]
*γ*	Recovered/Removed rate of the infectious	Days^-1^	0.2	0.1111–0.3333	[27–31]
*γ*'	Recovered/Removed rate of the asymptomatic	Days^-1^	0.1	0.04762–1	[32]
*f*_*i*_	Fatality of the disease of age group *i*	1	–	0–1	Analysis of data
*δ*	Vaccination rate	Days^-1^	–	0–1	Assumption
*φ*	Immune relative rate	1	–	0–1	[33–37]
*λ*	Vaccine efficacy	1	–	0–1	[38–50]
*θ*	Proportion of herd immunity	1	0	0–1	Assumption
*x*	Decreasing proportion of vaccine efficacy against susceptibility	1	0.6	0–1	[18]
*y*	Decreasing proportion of vaccine efficacy against infectivity	1	0.6	0–1	[18]
*z*	Decreasing proportion of vaccine efficacy against pathogenicity	1	0.33	0–1	[18]

– Not applicable

**Fig. 6:**
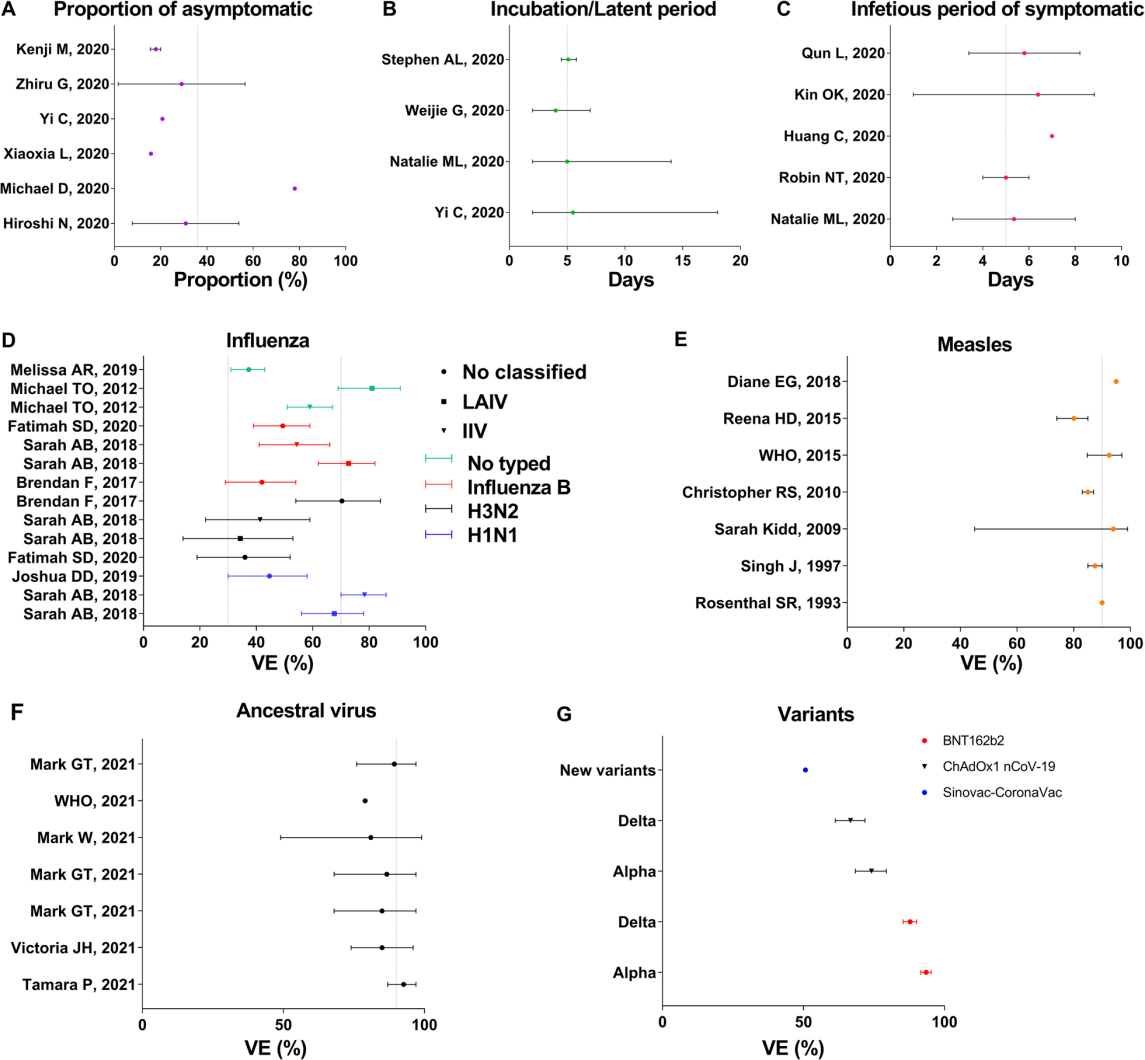
Summary of reported parameters of model about natural history and vaccination. **A** Proportion of asymptomatic (*p*). **B** Incubation period (*ω*) and latent period (*ω*’). **C** Infectious period of symptomatic (*γ*). **D** VE of influenza. **E** VE of measles. **F** VE after two doses vaccinating for the ancestral virus of COVID-19. **G** VE after two doses vaccinating for new variants of COVID-19

Several parameters are summarized in Fig. 6. The proportion of asymptomatic cases in the Diamond Princess cruise ship was 17.9% (95% confidence interval [*CI*]: 15.5–20.2%) and a study estimated the asymptomatic ratio as 30.8% (95% *CI*: 7.7–53.8%), by binomial distribution [23, 24]. Meanwhile, the asymptomatic proportion was reportedly 20.75% in Ningbo City, while another study indicated that it was much greater (78%). Therefore, we set the asymptomatic proportion (*p*) to 0.36 in the model (Fig. 6A).

According to published research, the incubation period during the early epidemic was 4 days (interquartile range: 2–7) in Wuhan City [25] and 5.1 days (95% *CI*: 4.5–5.8), based on publicly reported data [26]. Still, there is a wide range of incubation periods reported including incubation period in Ningbo City (2–18) and 95% *CI* in Wuhan City (2–14) [20, 27]. In the model, the incubation period was set to 5 days (*ω* = *ω*’ = 0.2).

In this study, it was assumed that the diagnosed cases would be immediately removed from the population. In the epidemiological characteristics of COVID-19 in Hong Kong, the period from symptom onset to confirmed diagnosis was 6.39 days (range: 1–8.83) [28]. The right truncation data indicated that the time from illness onset to hospitalization ranged from 2.7 to 8 days [27]. Meanwhile, other studies reported various findings regarding the time from symptom onset to hospitalization was, such as 7 days, 4–6 days, and 4.1–7.5 days [29–31]. In this model (Fig. 6C), the infectious period was set to 5 days (*γ* = 0.2). However, another study indicated that the median communicable period of 24 asymptomatic cases was 9.5 (range: 1–21) days [32]. We set *γ’* to 0.1 in the current model.

In Wuhan City, the total population was set to 11 080 996 (≤ 14 years: 1 256 552; 15–44 years: 5 210 885; 45–64 years: 3 374 388; ≥ 65 years: 1 239 171) for modelling purposes; according to the data analyses, the case fatality rate (*f*) was set to 0.1681% for age group 1, 0.5490% for age group 2, 3.4168% for age group 3, and 14.8424% for age group 4.

In the model, the vaccination rate (*δ*) was set to 0.00001, 0.0001, 0.001, 0.01 0.02, 0.03, 0.04, 0.05, 0.06, 0.07, 0.08, 0.09, and 0.1 based on the assumption of vaccination priority. In addition, we assumed that the effects of the COVID-19 vaccine would be similar to those of influenza A, influenza B, or measles vaccine in the scenario I. Most studies indicated that the time of development of influenza immunity ranged between 7 and 14 days [33–35]. Meanwhile, the highest rate of immunoglobulin G positivity in measles occurred in weeks 4 and 5 post-vaccination [36, 37]. In the model, immunity was set to 30 days after measles vaccination. Therefore, according to the effect of influenza A, influenza B, and measles, we set the immune relative rate (*φ*) to 1/7, 1/14, and 1/30, respectively. In addition, we simulated a scene to explore the conditions in which *φ* was equal to 1/5, 1/15, 1/20, 1/25, and 1/30, respectively. Several studies have reported that the VE of influenza ranged from 19 to 91%, with an especially wide range for influenza A (Fig. 6-D) [38–42]. In contrast, the VE of measles was reported to range from 45 to 99%, and the median was approximately 90% (Fig. 6-E) [43–50]. In this study, VE (*λ*) was set to 0.3, 0.7, and 0.9. Meanwhile, a scene based on different VE was simulated in the model, set *λ* to 0, 0.1, 0.2, …, 0.9, and 1. Thereafter, the parameters of scenario II were collected from a study that indicated VE_*S*_ = 0.4, VE_*I*_ = 0.4, and VE_*P*_ = 0.67. In the model, *x* = 0.6, *y* = 0.6, and *z* = 0.33. Meanwhile, the immune relative rate (*φ*) was set to 0.1 in scenario II, according to the vaccine effect of H1N1.

Most studies have reported that the mean value of VE was more than 90% (with two-dose administration) for controlling ancestral virus (Fig. 6-F) [5–7, 9, 51], 68%–95% (with two-dose administration) for controlling infection of the Alpha variant, and more than 62% (with two-dose administration) for controlling infection of the Delta variant (Fig. 6G) [8].

### Estimated transmission

In this study, we adopted two indicators to estimate the transmissibility and risk of infection, including the effective reproduction number (*R*_eff_, the mean number of secondary cases an infected person can cause in a population after implementation of intervention measures), and the probability of infection from a single contact (*q*, the possibility of the susceptible person becoming infected after effective contact).

The equation of *R*_eff_ is as follows:

$${R}_{\mathrm{eff}}= \beta N(\frac{1-p}{\gamma }+\frac{\kappa p}{{\gamma }^{^{\prime}}})$$

The equation of *q* is as follows:

$$\beta N= 1-{(1-q)}^{\alpha }$$

In the above equation, *α* is defined as the contact frequency per day, which was calculated from a previously published paper [52].

To compare the transmission in different areas, we calculated the median of the normalized *R*_eff_, in four stages. The relative transmissibility in the different age groups was quantified by an equation, and the min–max normalized (the lower and upper bounds of relative transmissibility) version was used:

$${Normalized~R}_{\mathrm{eff}}= \frac{x-\mathrm{min}(x)}{\mathrm{max}\left(x\right)-\mathrm{min}(x)}$$

*x* is the value of *R*_eff-*ij*_ (subscript *i* and *j* (*i* ≠ *j*) equals 1 to 4, respectively). Thereafter, we further compared the normalized results of Wuhan City, Hunan Province, and Jilin Province to explain the heterogeneity of age-related transmission in different areas. The results of the above provinces were from a previously published paper [12]; we re-calculated *R*_eff_ according to the parameter *β* results.

To explore transmission interactions (infected and infected by others) in the four age groups, we adopted two indicators to estimate the infectivity and susceptibility of each group in four stages. *R*_I_ indicated infectivity, and *R*_s_ was the susceptibility in a specific age group, which was calculated as follows:

$${R}_{\mathrm{I}-i}= \sum_{j=1}^{n}{R}_{\mathrm{eff}-ij}$$

$${R}_{\mathrm{S}-i}=\sum_{j=1}^{n}{R}_{\mathrm{eff}-ji}$$

In the above equation, *n* is equal to four. For example, *the R*_I_ of age group 1 is the sum of *R*_eff-11_, *R*_eff-12_, *R*_eff-13_, and *R*_eff-14_, and the *R*_S_ of age group 1 is the sum of *R*_eff-11_, *R*_eff-21_, *R*_eff-31_, and *R*_eff-41_.

### Vaccine effectiveness

We evaluated the vaccination effects for controlling transmission and disease severity.

The transmission was estimated using six indicators, including the total number of new cases (*TN*), total attack rate (*TAR*), and the number of new cases at peak (*NP*), and two positive indicators, duration of outbreak (*DO*) and peak time (*PT*). The equation is as follows:

$$TN= Total~number~of~new~cases$$

$$TAR= \frac{TN}{N} \times 100\%$$

$$DO= {t}_{0}-{t}_{1}$$

$$PT= {t}_{p}$$

$$NP= Number~of~new~cases~at~peak$$

In the above equation, *N*, *t*_0_, *t*_1_, and *t*_*p*_ refer to the number of total population, illness onset date of the first case, illness onset date of the last case, and the peak of the infection curve, respectively.

The disease severity was estimated based on the total number of deaths (*ND*). The equation is as follows:

$$ND= Total~number~of~death$$

Because of the competitive relationship between vaccination rate (*δ*) and the relative transmission relative (*β*), we cannot directly consider vaccination rate as vaccination coverage. In the model, we assumed that the start time of vaccination was *t*_*s*_, and the end time (the vaccination time of the last susceptible person) was *t*_*e*_. We calculated the vaccination coverage for the total population and each age group. The vaccination coverage for each day was calculated from the differential equation of the vaccination model. Model 2 was calculated as follows:

$$Vaccination~coverage (n)=\frac{{\delta }_{i}}{{\beta }_{ii}\left({I}_{i}+\kappa {A}_{i}\right)+{\beta }_{ji}\left({I}_{j}+\kappa {A}_{j}\right)+{\delta }_{i}}$$

Model 3 was calculated as follows:

$$Vaccination~coverage (n)={{\delta }_{i}/(\beta }_{ii}{((I}_{1i}+\kappa {A}_{1i})+{y(I}_{2i}+\kappa {A}_{2i}))+{\beta }_{ji}{((I}_{1j}+\kappa {A}_{1j})+{y(I}_{2j}+\kappa {A}_{2j}))+{\beta }_{ii}{\theta }_{i}(x({I}_{1i}+\kappa {A}_{1i})+z({I}_{2i}+\kappa {A}_{2i})){ + \beta }_{ji}{\theta }_{i}(x({I}_{1j}+\kappa {A}_{1j})+z({I}_{2j}+\kappa {A}_{2j}))+{\delta }_{i})$$

*n* refers to vaccination coverage of *n*th day. Thereafter, the total vaccination coverage was calculated by integrating vaccination coverage of the vaccination period (from *t*_*s*_ to *t*_*e*_). The equation is as follows:

$$Total~vaccination~coverage={\int }_{{t}_{s}}^{{t}_{e}}vaccination~coverage \left(t\right) dt$$

In the equation, *t*_*s*_ and *t*_*e*_ refer to the start time of the vaccination period and the end time (the vaccination time of the last susceptible person), respectively.

### Simulation and statistical analysis

**Fig. 7:**
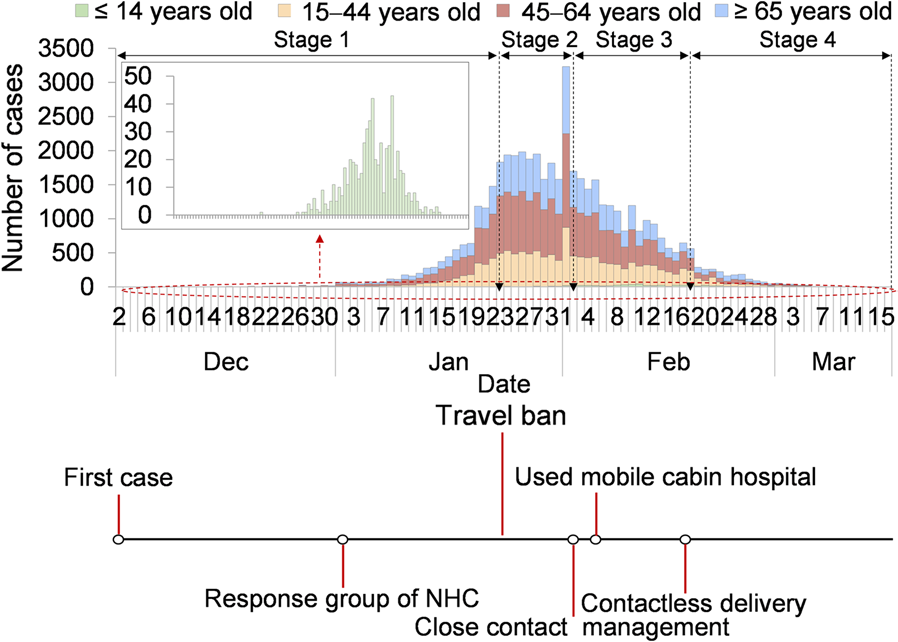
Epidemic curve of four age-group and key interventions in Wuhan City. **A** Epidemic curve. **B** Intervention measures

According to previously published studies [53–55], we assumed that heterogeneity of transmissibility existed as an ascending trend and then a descending trend. The data were divided into several segments (defined as stages); for example, Wuhan City was divided into four stages in the disease transmission period (Fig. 7). The period of each stage was as follows:

- Stage 1 refers to the illness onset date from December 2, 2019, to January 23, 2020.
- Stage 2 refers to the illness onset date from January 24 to February 2, 2020.
- Stage 3 refers to the illness onset date from February 3 to February 18, 2020.
- Stage 4 refers to the illness onset date from February 19, 2020, to March 16, 2020.

Curve fitting and model simulation adopted the least root-mean-square deviations. The simulation method used the Runge–Kutta method of order four with a tolerance set to 0.001. The differential equations were solved in steps of 0.02 days. The goodness of fit was judged by the coefficient of determination (*R*^2^).

## Results

### Epidemiological characteristics and model effectiveness

From December 2, 2019, to March 16, 2020, a total of 47 722 new cases (cumulative incidence: 431 cases per 10 000 persons) were reported in Wuhan City (Fig. 7). In those ≤ 14 years old, there were 595 reported cases (cumulative incidence: 47 cases per 10 000 persons); 12 933 cases were reported in 15–44-year-olds (cumulative incidence: 248 cases per 10 000 persons), 20,106 cases reported among 45–64-year-olds (cumulative incidence: 596 cases per 10 000 persons) and 14,088 cases reported among those ≥ 65 years old (cumulative incidence: 1 134 cases per 10 000 persons).

The date of the first case onset was on December 2, 2019. The National Health Commission of China set up a response group on January 1, 2020. Thereafter, several intervention measures were adopted by Wuhan City, such as travel ban (January 23, 2020), close-contact management (February 3, 2020), mobile cabin hospitals (February 5, 2020), and contactless delivery (February 17, 2020).

**Fig. 8:**
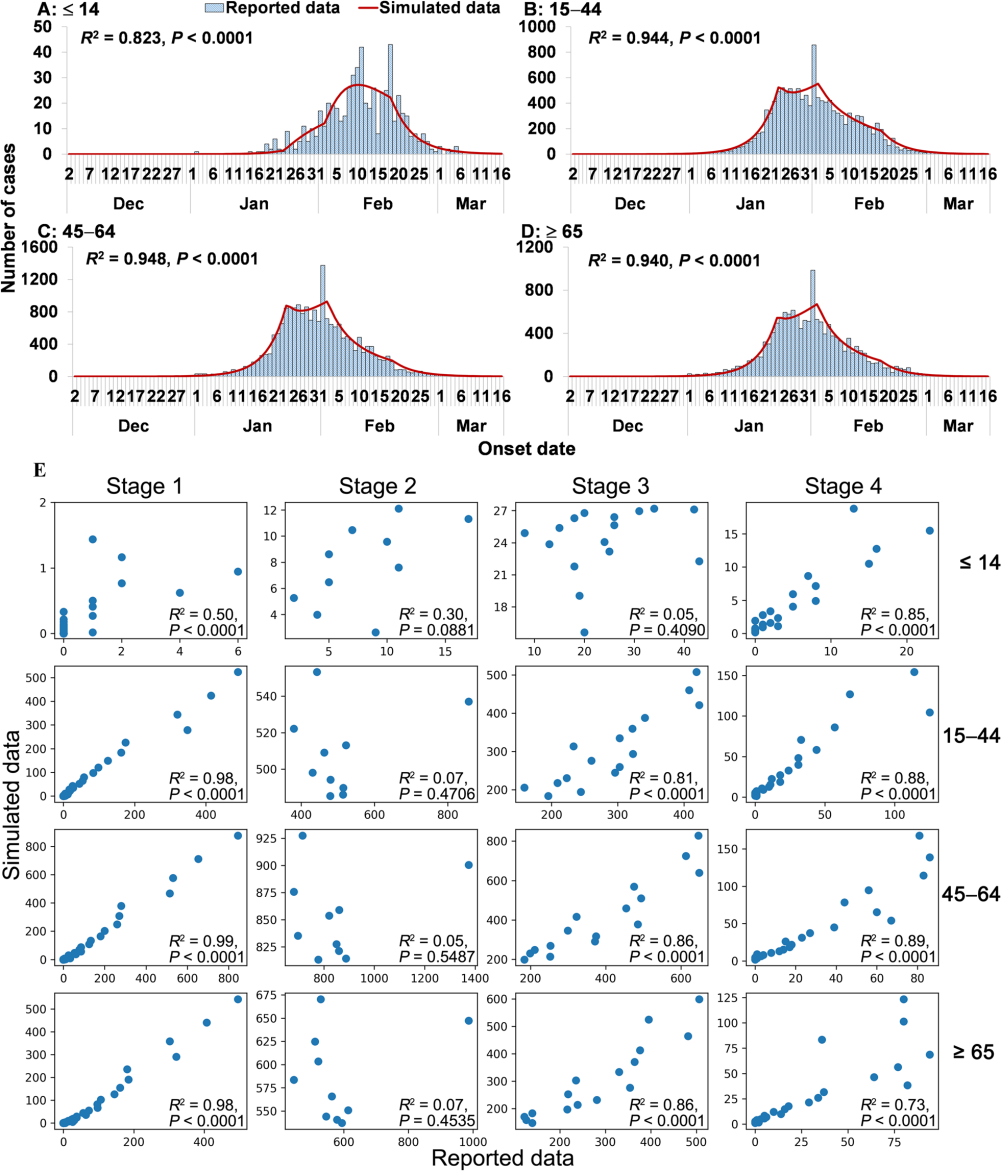
Curve fitting of the age-specific SEIAR model to the reported data in Wuhan City. **A** ≤ 14 years. **B** 15‒44 years. **C** 45‒64 years; **D** ≥ 65 years. **E** Fitting results in four stages of four age groups

In Wuhan City, the age-specific model (Fig. 8) fits the total reported data for the four age groups most effectively (≤ 14 years: *R*^2^ = 0.823, *P* < 0.0001; 15–44 years: *R*^2^ = 0.944, *P* < 0.0001; 45–64 years: *R*^2^ = 0.948, *P* < 0.0001; ≥ 65 years: *R*^2^ = 0.940, *P* < 0.0001). However, the model fits the reported data in stage 2 and is not significant in stage 3 in those ≤ 14 years of age.

### Transmissibility and risk of infection

**Fig. 9:**
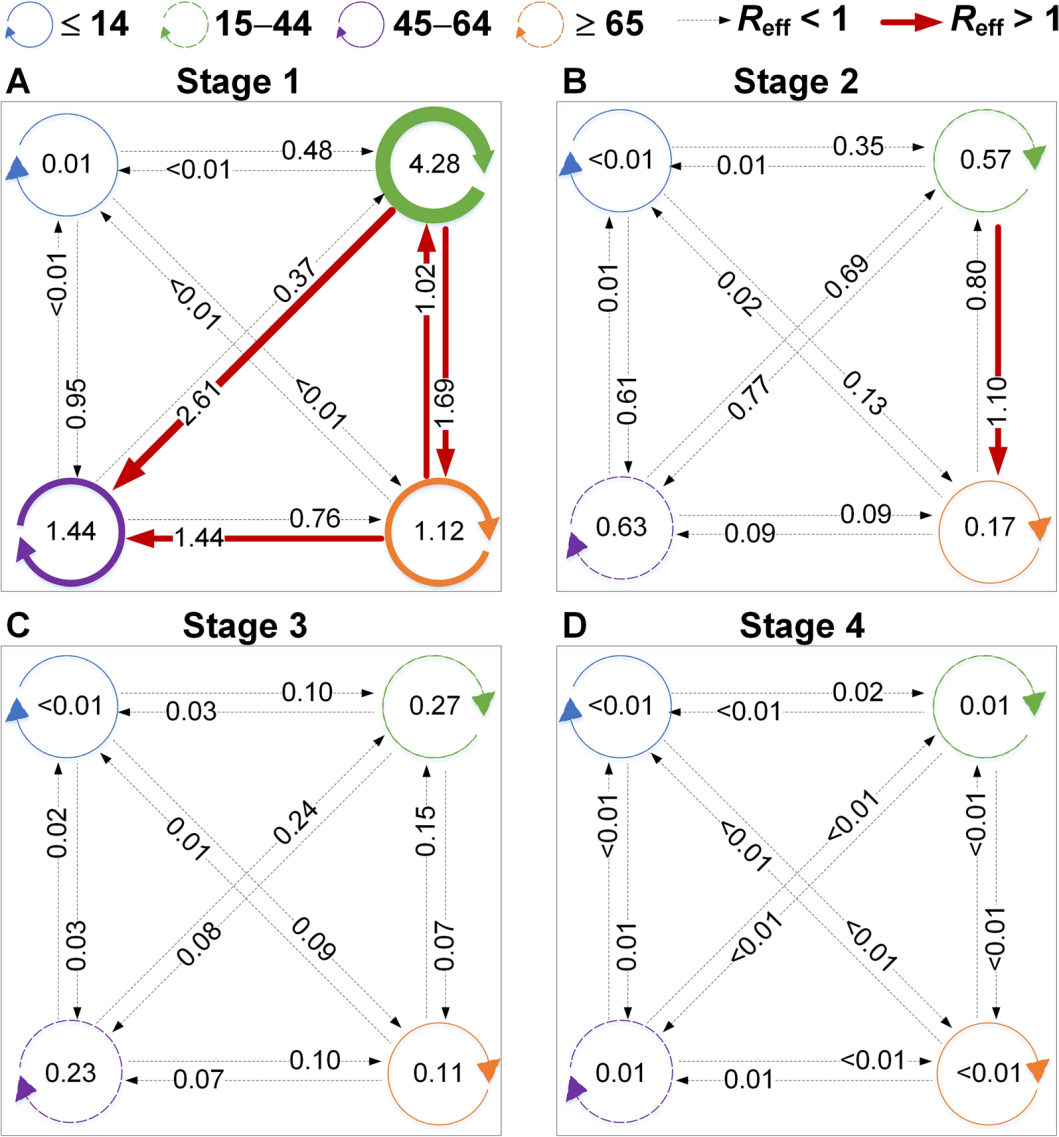
The interaction of transmissibility between different age groups in four stages of Wuhan City. **A** Stage 1: December 2, 2019, to January 23, 2020. **B** Stage 2: January 24 to February 2, 2020. **C** Stage 3: February 3 to February 18, 2020. **D** Stage 4: February 19, 2020, to March 16, 2020

According to the simulated results (Fig. 9), in stage 1, the highest transmissibility occurred among the members of age group 2 (*β*_22_ = 1.50 × 10^-7^, *R*_eff-22_ = 4.28), followed by age group 2 to 3 (*β*_23_ = 1.57 × 10^-7^, *R*_eff-23_ = 2.61), age group 2 to 4 (*β*_24_ = 3.26 × 10^-7^, *R*_eff-24_ = 1.69), among the members of group 3 (*β*_33_ = 8.43 × 10^-8^, *R*_eff-33_ = 1.44), and age group 4 to 3 (*β*_43_ = 8.39 × 10^-8^, *R*_eff-43_ = 1.44). In stage 2, the highest transmissibility occurred from age group 2 to 4 (*β*_24_ = 2.12 × 10^-7^, *R*_eff-24_ = 1.10), while the other group combinations had a *R*_eff_ value lower than 1. Thereafter, all the values of *R*_eff_ were lower than 1 in stages 3 and 4. Our results suggest that transmission was effectively controlled after travel ban in Wuhan City.

**Fig. 10:**
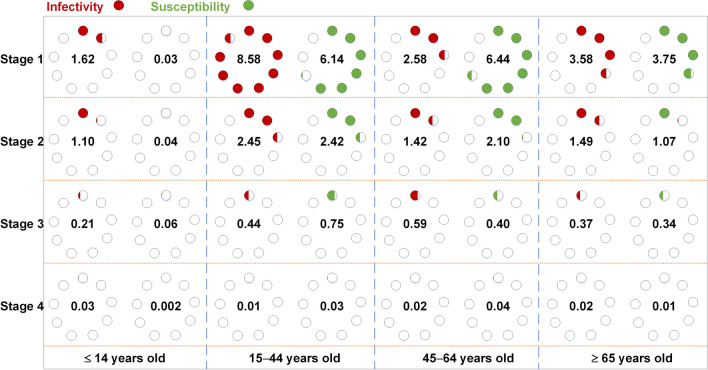
The infectivity and susceptibility of different age groups in four stages of Wuhan City

**Fig. 11:**
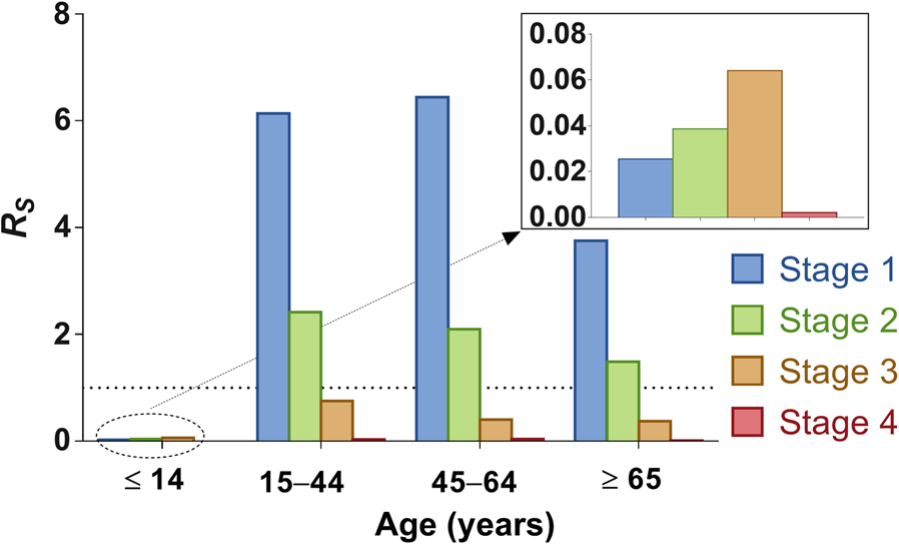
The susceptibility comparison of different age groups in four stages of Wuhan City

In stage 1, the highest infectivity was in age group 2 (*R*_I_ = 8.58), followed by age group 4 (*R*_I_ = 3.58), age group 3 (*R*_I_ = 2.58), and age group 1 (*R*_I_ = 1.62). The highest susceptibility was observed in age group 3 (*R*_S_ = 6.44), followed by age group 2 (*R*_S_ = 6.14), age group 4 (*R*_S_ = 3.75), and age group 1 (*R*_S_ = 0.03). In stage 2, the infectivity and susceptibility were as follows: in age group 1 (*R*_I_ = 1.10, *R*_S_ = 0.04), age group 2 (*R*_I_ = 2.45, *R*_S_ = 2.42), age group 3 (*R*_I_ = 1.42, *R*_S_ = 2.10), and age group 4 (*R*_I_ = 1.49, *R*_S_ = 1.07), respectively. The values of *R*_I_ and *R*_S_ were all lower than 1 in stages 3 and 4 (Fig. 10). In addition, susceptibility tended to increase in age group 1 from stage 1 to 3 (Fig. 11).

**Fig. 12:**
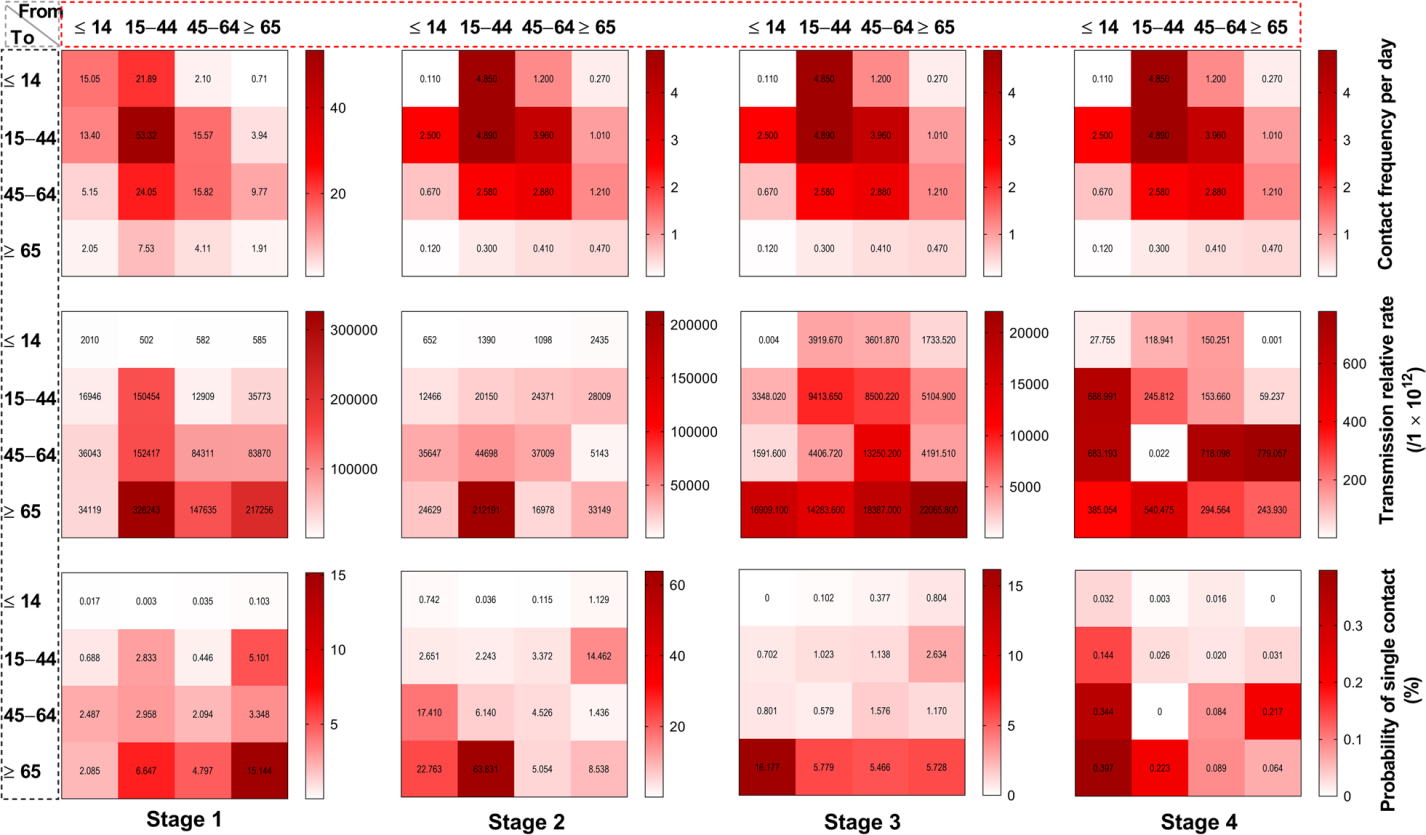
The risk of infected probability in different age groups of four stages

The highest contact frequency in stage 1 occurred among the members of age group 2 (*α* = 53.32), followed by age group 2 to 3 (*α* = 24.05), age group 2 to 1 (*α* = 21.89), and among the members of age group 3 (*α* = 15.82). In the elderly, we found the highest probability of infection to be from a single contact (Fig. 12). When considering the probability of infection from a single contact, the highest value in stage 1 occurred among the members of age group 2 (*q* = 15.144%), followed by age group 2 to 4 (*q* = 6.647%), age group 4 to 2 (*q* = 5.101%), and age group 3 to 4 (*q* = 4.797%). In stage 2, the highest probability of infection from a single contact was occurred from age group 2 to 4 (*q* = 63.831%), followed by age group 1 to 4 (*q* = 22.763%), age group 1 to 3 (*q* = 17.410%), and age group 4 to 2 (*q* = 14.462%). In stage 3, the highest probability of infection from a single contact was found in age group 1 to 4 (*q* = 16.177%), followed by age group 2 to 4 (*q* = 5.779%), among the members of age group 4 (*q* = 5.728%), and age group 3 to 4 (*q* = 5.466%). In stage 4, the highest value was found in age group 1 to 4 (*q* = 0.397%), followed by age group 1 to 3 (*q* = 0.344%), age group 2 to 4 (*q* = 0.223%), and age group 4 to 3 (*q* = 0.217%).

**Fig. 13:**
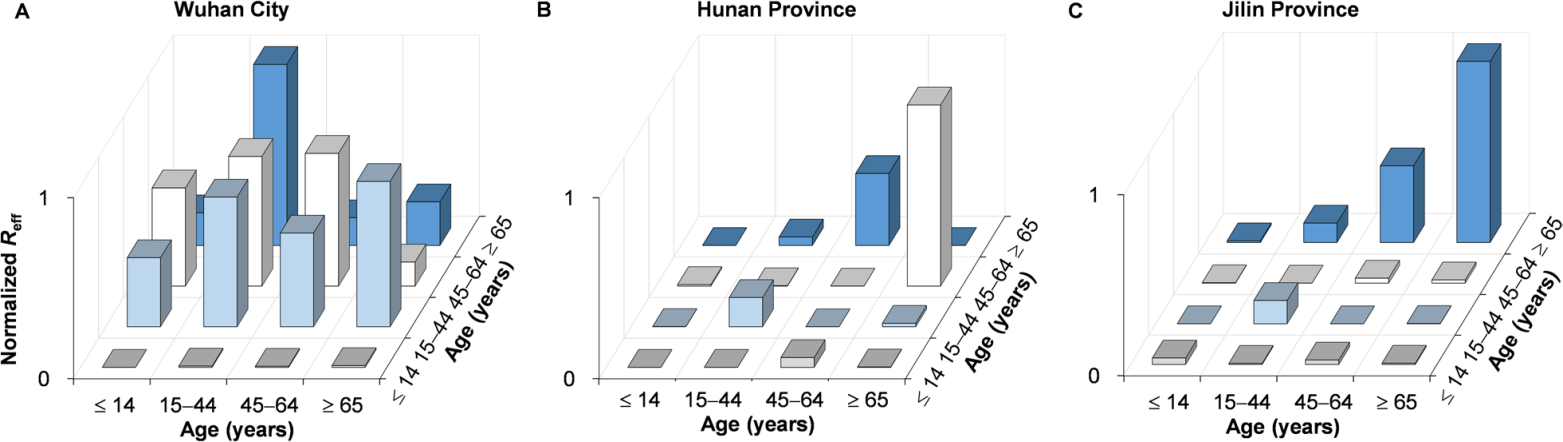
The relative transmissibility of different age groups in four stages of Wuhan City, Hunan Province, and Jilin Province. **A** Normalized *R*_eff_ of Wuhan City. **B** Normalized *R*_eff_ of Hunan Province. **C** Normalized *R*_eff_ of Jilin Province. **B**, **C** were a re-calculated value of *R*_eff_ from the matrix of secondary attack rate of our previous study (https://doi.org/10.1186/s40249-020-00735-x)

We found differing age-specific transmission patterns in several areas (Fig. 13). The highest relative transmissibility in Wuhan City was from age group 2 to 4, followed by group 4 to 2, 3 to 3, and 2 to 3. In Hunan Province, the highest relative transmissibility was observed in age group 4 to 3, followed by group 3 to 4, 2 to 2, and 3 to 1. In Jilin Province, it was observed from age group 4 to 4, followed by group 3 to 4, 2 to 2, and 2 to 4.

### Effectiveness of vaccination against transmission

**Fig. 14:**
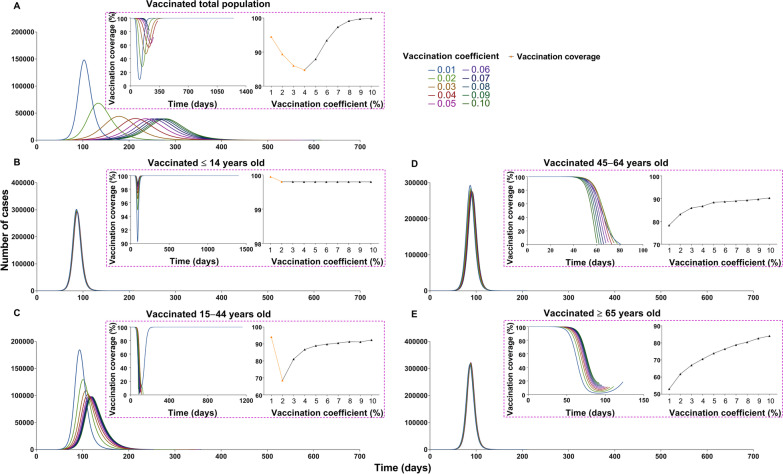
The influence of different vaccination coverages from each age group to total population in scene XI of scenario I. **A** Number of cases in total population when vaccinated the total population. **B** The number of cases in total population when vaccinated ≤ 14 years old. **C** The number of cases in total population when vaccinated 15‒44 years old. **D** The number of cases in total population when vaccinated 45‒64 years old. **E** Number of cases in total population when vaccinated ≥ 65 years old

**Fig. 15:**
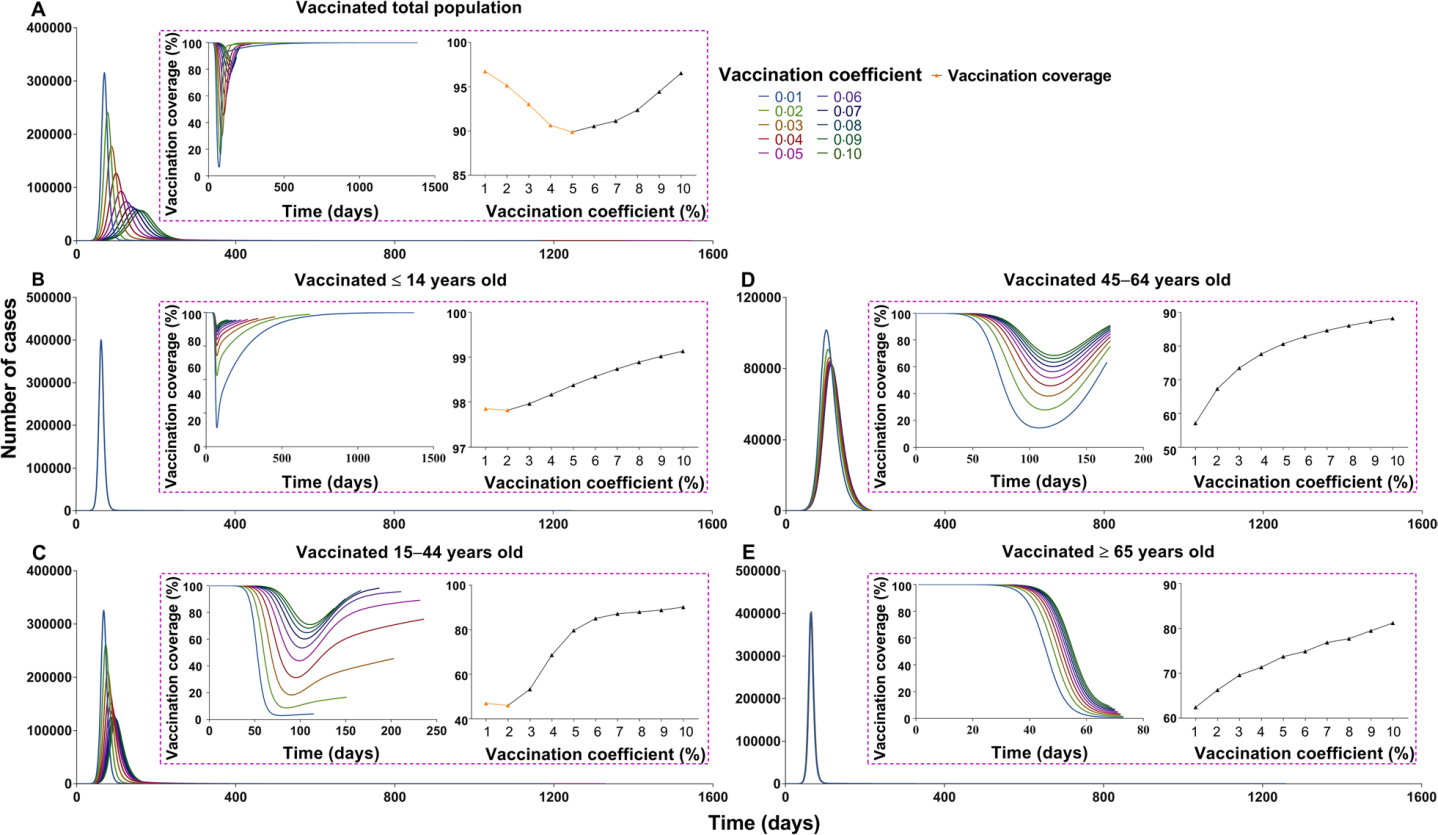
The influence of different vaccination coverages from each age group to total population in scene XIX of scenario II. **A** The number of cases in total population when vaccinated the total population. **B** The number of cases in total population when vaccinated ≤ 14 years old. **C** The number of cases in total population when vaccinated 15‒44 years old. **D** The number of cases in total population when vaccinated 45‒64 years old. **E** Number of cases in total population when vaccinated ≥ 65 years old

**Fig. 16:**
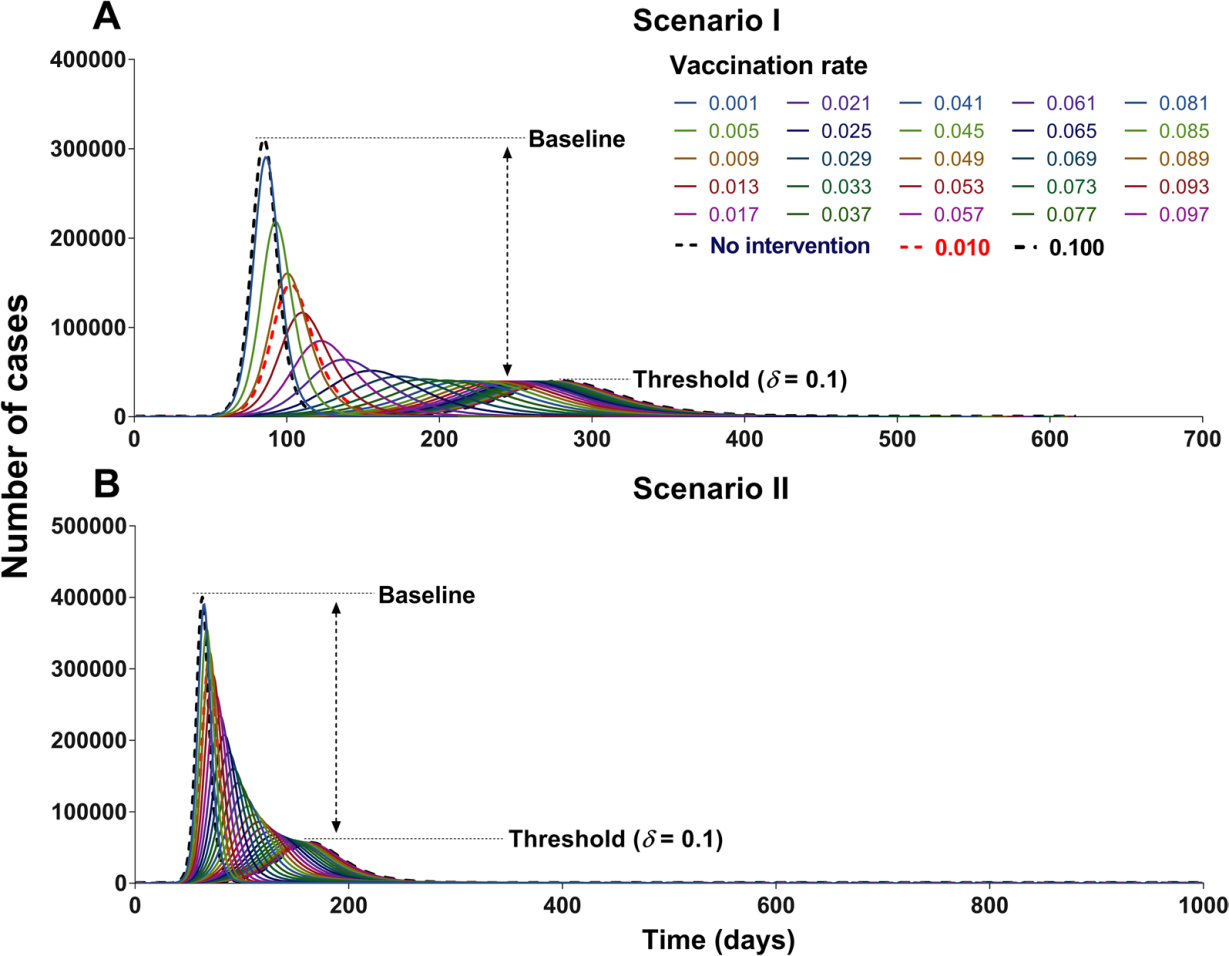
Simulated the effects of different vaccination rates in scene I of scenario I and scene XI scenario II. **A** Different vaccination rates of scenarios I. **B** Different vaccination rates of scenarios II

**Fig. 17:**
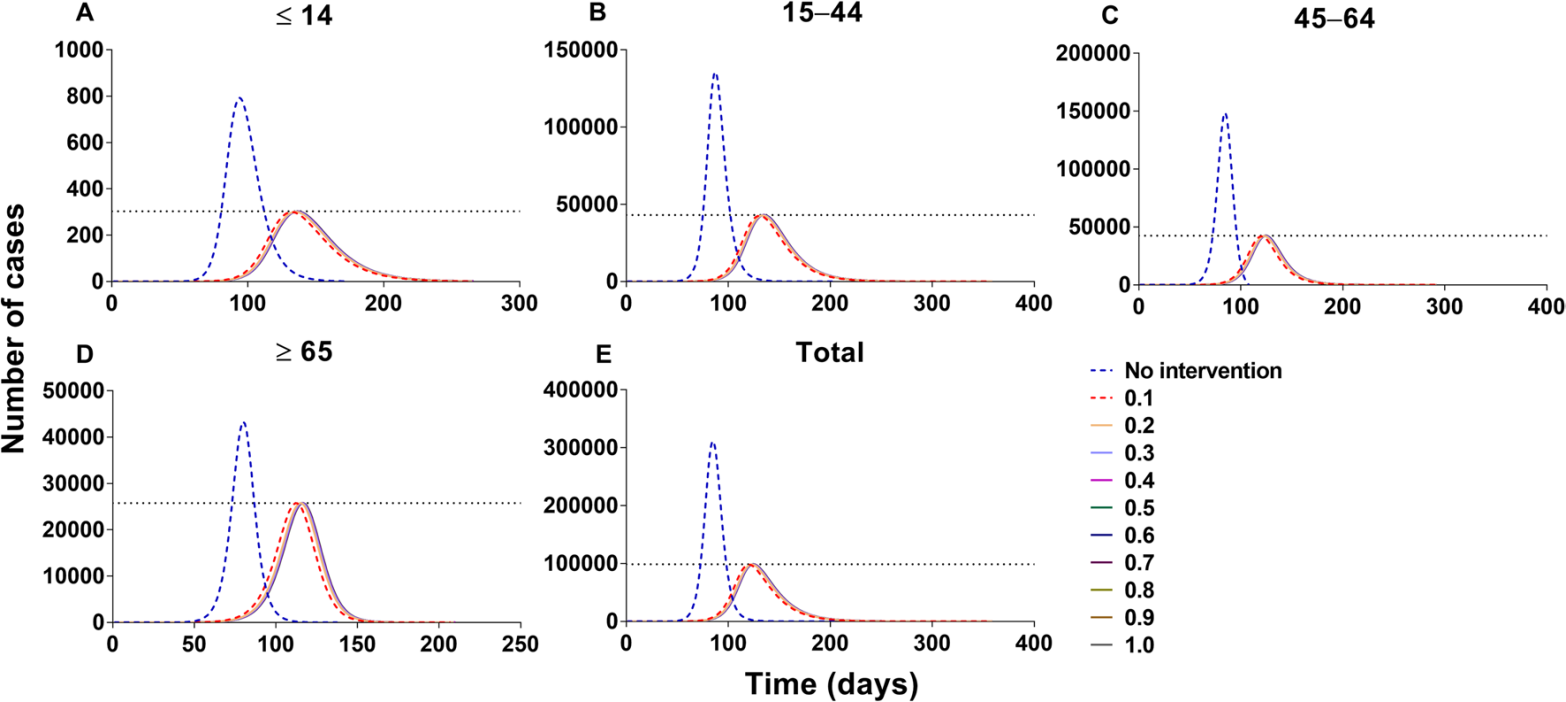
Simulated the different vaccination rates in each age group. **A** Vaccinated individuals ≤ 14 years old. **B** Vaccinated individuals 15‒44 years old. **C** Vaccinated individuals 45‒64 years old. **D** Vaccinated individuals ≥ 65 years old. **E** Vaccinated total population

China should vaccinate at least 85% of the total population to interrupt transmission, with a VE of more than 70% (Figs. 14 and 15). The vaccine effects increased with an increase in vaccination rate (Fig. 16). However, it reached a threshold (Fig. 17) when *δ*_*i*_ = 0.1, regardless of scenario I (*TN* = 4 722, *TAR* = 0.04%, *DO* = 177, *PT* = 76, *NP* = 68) or scenario II (*TN* = 4 548 464, *TAR* = 41.05%, *DO* = 997, *PT* = 162, *NP* = 56 613).

In scene XI of scenario I (Fig. 14), the epidemic was controlled when vaccinating 87.93% of the whole population (*δ*_*i*_ = 0.05, *NT* = 4 028 228, *TAR* = 36.35%, *DO* = 574, *PT* = 236, *NP* = 39 648). The optimal strategy was first to vaccinate 88.96% (Additional file 4: Table S3) of individuals 15–44 years old (*δ*_*2*_ = 0.05, *NT* = 4 780 964, *TAR* = 43.15%, *DO* = 351, *PT* = 114, *NP* = 99 288) and then to vaccinate 90.28% of individuals 45–64 years old (*δ*_*3*_ = 0.1, *NT* = 6 329 126, *TAR* = 57.12%, *DO* = 211, *PT* = 92, *NP* = 274 421). In scene XIX of scenario II (Fig. 15), the epidemic was controlled when vaccinating 96.53% of the whole population (*δ*_*i*_ = 0.1, *NT* = 4 548 464, *TAR* = 41.05%, *DO* = 997, *PT* = 162, *NP* = 56 613). The optimal vaccination strategy was first to vaccinate 90.18% of individuals 15–44 years old (*δ*_*2*_ = 0.1, *NT* = 5 189 445, *TAR* = 46.83%, *DO* = 188, *PT* = 98, *NP* = 121 696) and secondly to vaccinate 88.21% of individuals aged 45–64 years old (*δ*_*3*_ = 0.1, *NT* = 4 717 545, *TAR* = 42.57%, *DO* = 217, *PT* = 114, *NP* = 82 318). Our findings suggest that the vaccination priority for controlling the transmission may be vaccinating approximately 90% of individuals aged between 15 and 44 years old.

**Fig. 18:**
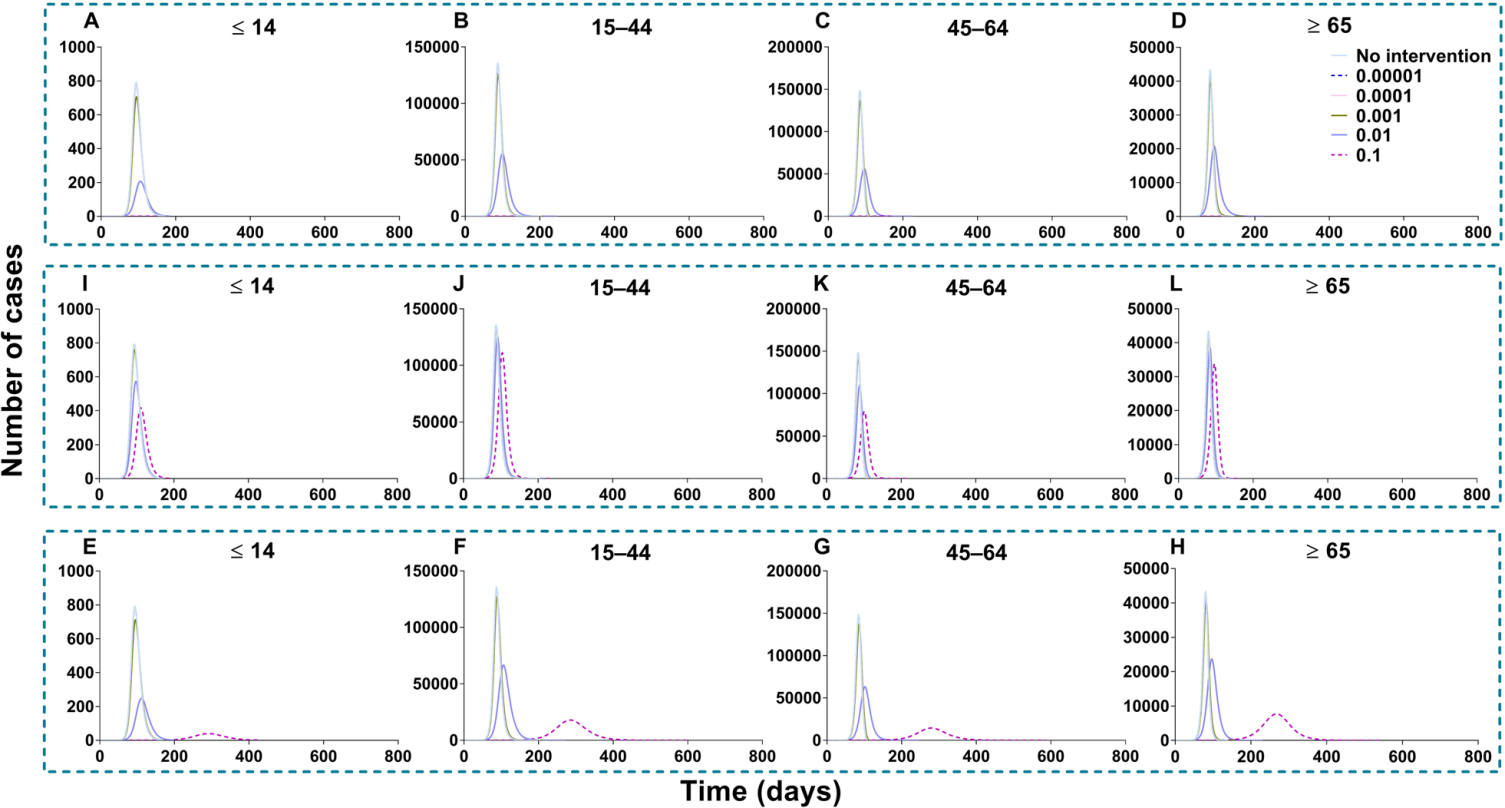
Simulated the vaccine effects same as measles and influenza vaccine in scene II, III, and IV of scenario I. **A** Measles vaccine vaccinated total population (VE = 90%). **B** High VE of influenza vaccine vaccinated total population (VE = 70%). **C** Low VE of influenza vaccine vaccinated total population (VE = 30%)

All the values of scenario I (scenes II to IX) are shown in Additional file 5: Tables S4 to S7. In scenario I of no intervention, the simulation of value was in the total population (*TN* = 6 776 654, *TAR* = 61.16%, *DO* = 205, *PT* = 84, *NP* = 311 342), in age group 1 (*TN* = 23 595, *TAR* = 1.88%, *DO* = 172, *PT* = 94, *NP* = 792), group 2 (*TN* = 3 136 946, *TAR* = 60.20%, *DO* = 205, *PT* = 87, *NP* = 135 616), group 3 (*TN* = 2 823 084, *TAR* = 83.66%, *DO* = 108, *PT* = 84, *NP* = 148 321) and group 4 (*TN* = 793 029, *TAR* = 64.00%, *DO* = 138, *PT* = 80, *NP* = 43 328). The simulation effects of vaccination in each age group were reduced with the increasing vaccination rate (*δ*), and the effects of scene II (measles vaccine) were higher than those of scenes III and IV (influenza vaccine). The best effect (Fig. 18), in which the total population was vaccinated (*δ*_*I*_ = 0.1), was scene II (*TN* = 4 722, *TAR* = 0.04%, *DO* = 177, *PT* = 76, *NP* = 68), followed by scene III (*TN* = 4 026 365, *TAR* = 36.34%, *DO* = 601, *PT* = 278, *NP* = 39 534) and then scene IV (*TN* = 6 127 075, *TAR* = 55.29%, *DO* = 232, *PT* = 101, *NP* = 214 987).

**Fig. 19:**
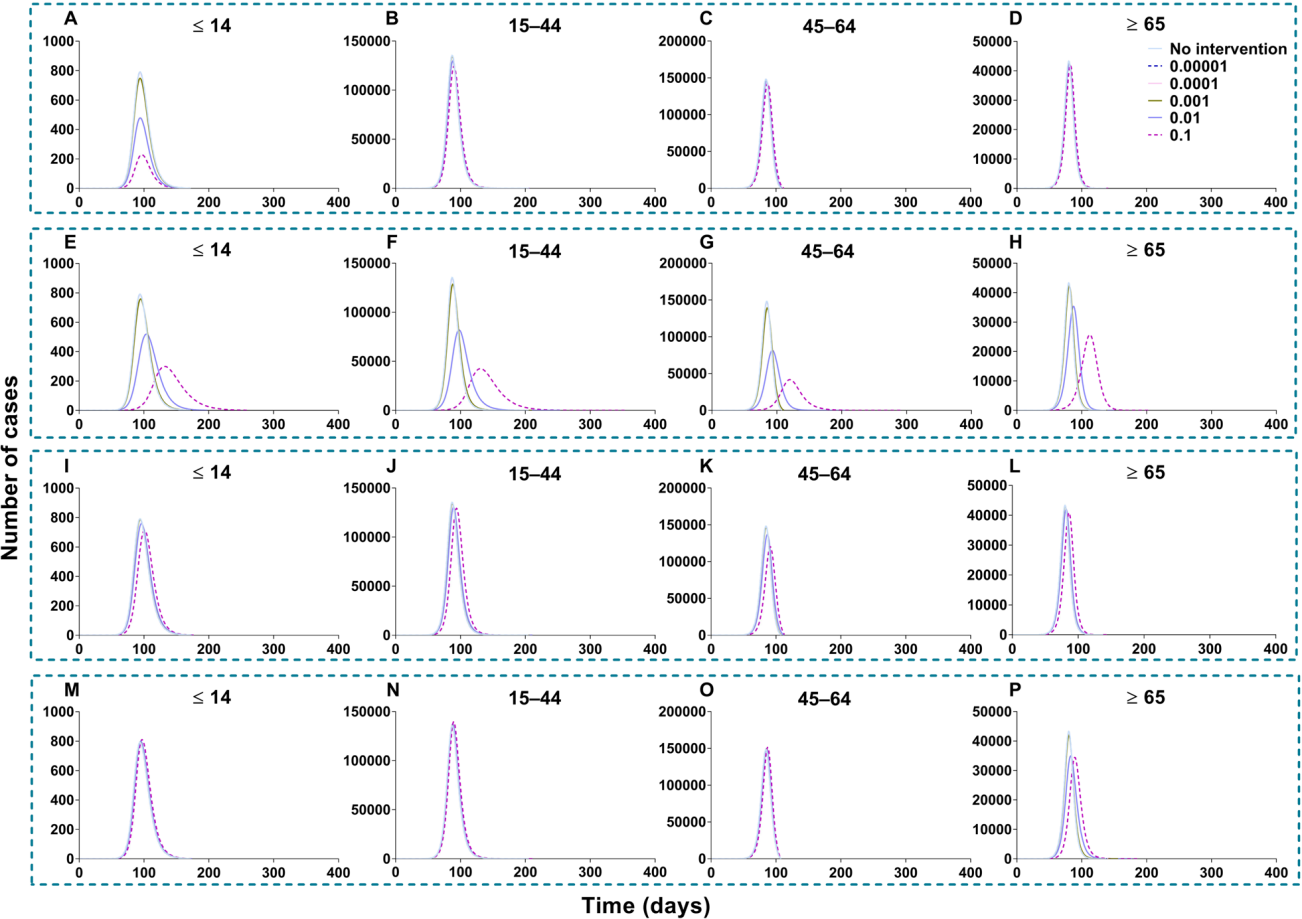
Simulated the effects vaccinated each age group in scene V, VI, VII, and VIII of scenario I. **A**‒**D** Vaccinated individuals ≤ 14 years old. **E**‒**H** Vaccinated individuals 15‒44 years old. **I**‒**L** Vaccinated individuals 45‒64 years old. **M**‒**P** Vaccinated individuals ≥ 65 years old

Scenes V, VI, VII, and VIII of scenario I are shown in Fig. 19, when the vaccination was simulated in each age group. The optimal strategy was to vaccinate age group 2 in the total population, especially when *δ*_*2*_ = 0.1 (*TN* = 4 802 585, *TAR* = 43.34%, *DO* = 357, *PT* = 119, *NP* = 97 964). After vaccination in age groups 1, 3, and 4, it was less affected than the other three groups. With vaccination in age group 1, the greatest effect (*δ*_*1*_ = 0.1) was only for itself (*TN* = 6 942, *TAR* = 0.55%, *DO* = 161, *PT* = 97, *NP* = 229). With vaccination in age group 2, the greatest effect (*δ*_*2*_ = 0.1) was only for itself (*TN* = 2 310 030, *TAR* = 44.33%, *DO* = 357, *PT* = 131, *NP* = 42 553). With vaccination in age group 3, the greatest effect (*δ*_*3*_ = 0.1) was only for itself (*TN* = 2 419 225, *TAR* = 71.69%, *DO* = 113, *PT* = 91, *NP* = 120 356). With vaccination in age group 4, the greatest effect (*δ*_*4*_ = 0.1) was only for itself (*TN* = 778 980, *TAR* = 62.86%, *DO* = 188, *PT* = 89, *NP* = 34 451).

**Fig. 20:**
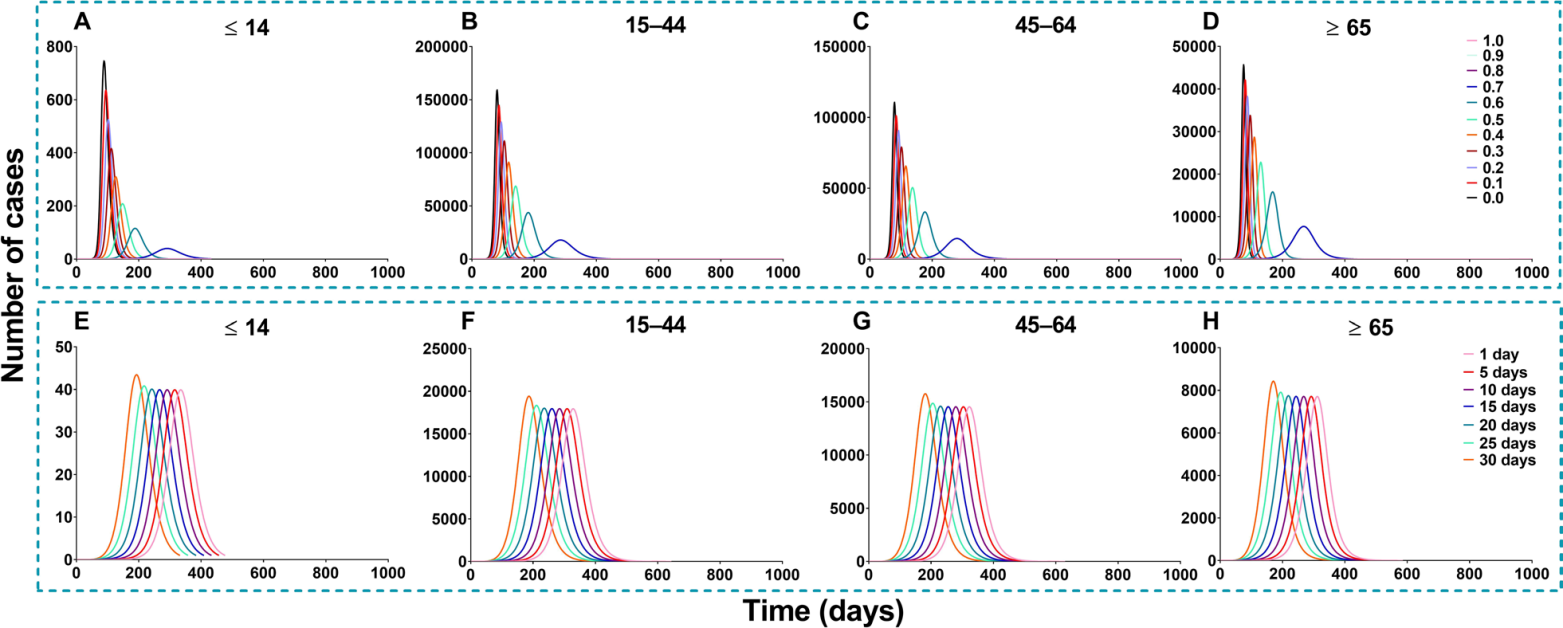
Simulated the different VE and immune relative rates in scenes IX and X of scenario I. **A**‒**D** Different VE vaccinated total population. **E**‒**H** With immunity after different periods vaccinated total population

The simulation of different VE and the relative immune rates in the total population is shown in Fig. 20. The best simulation effect of VE was *λ* = 1 and *λ* = 0.9 (*TN* = 0, *TAR* = 0, *DO* = 0, *PT* = 0, *NP* = 0), and followed by *λ* = 0.8 (*TN* = 531 227, *TAR* = 4.79%, *DO* = 3 804, *PT* = 1 862, *NP* = 455). The best simulation effect of the relative immune rate was *φ* = 1 (*TN* = 4 026 318, *TAR* = 36.34%, *DO* = 645, *PT* = 322, *NP* = 39 533) and the worst effect of the relative immune relative was *φ* = 0.033 (*TN* = 4 078 737, *TAR* = 36.81%, *DO* = 494, *PT* = 179, *NP* = 43 113).

**Fig. 21:**
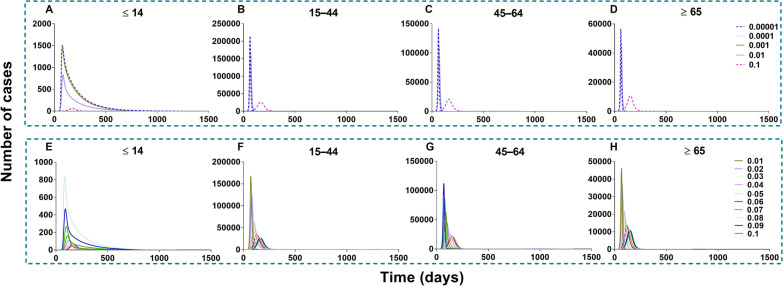
Simulated the different vaccination rates when vaccinated total population in scene XII of scenario II. **A**‒**D** Vaccination rate was 0.0001, 0.001, 0.01 and 0.1, respectively. The number of cases in the no-vaccinated group when vaccinated total population. **E**‒**H** Vaccination rate from 0.01 to 0.1

All the values of scenario II (scenes XI to XV) are shown in Additional file 6: Tables S8 to S11. Vaccination of the total population in scenario II (Fig. 21), the total number of cases was reduced by the rising vaccination rates. The best simulation effect was when *δ*_*i*_ = 0.1 for the number of cases in total population (*TN* = 4 548 464, *TAR* = 41.05%, *DO* = 997, *PT* = 162, *NP* = 56 613), respectively.

**Fig. 22:**
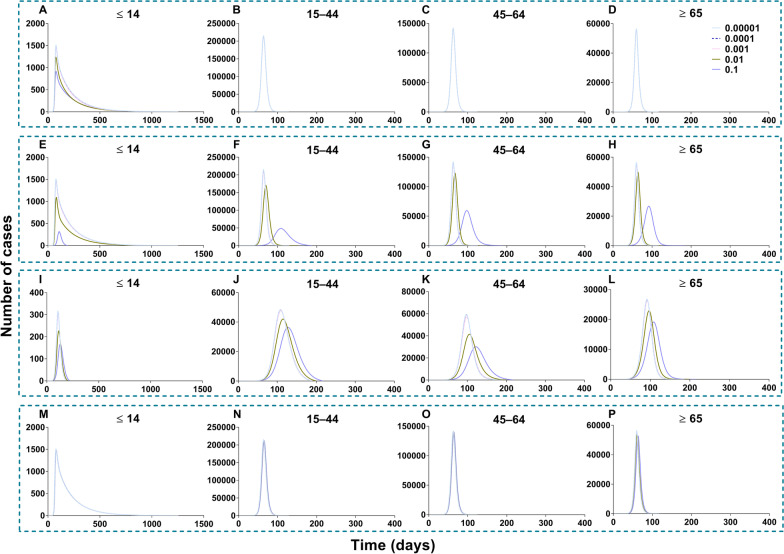
Simulated the different vaccination rates in each age group in scenes XI, XII, XIII, and XIV of scenario II. **A**‒**D** Vaccinated individuals ≤ 14 years old. **E**‒**H** Vaccinated individuals 15‒44 years old. I‒**L** Vaccinated individuals 45‒64 years old. **M**‒**P** Vaccinated individuals ≥ 65 years old

Regarding the vaccination of each age group in scenario II (Fig. 22), the optimal strategy was vaccination of age group 2 for the total population, especially when *δ*_*2*_ = 0.1 (*TN* = 5 189 459, *TAR* = 46.83%, *DO* = 190, *PT* = 97, *NP* = 121 696) and age group 3, especially when *δ*_*3*_ = 0.1 for the total population (*TN* = 4 624 965, *TAR* = 41.74%, *DO* = 219, *PT* = 97, *NP* = 378 275). Less effects were found with vaccinated age group 1 (*TN* = 6 429 821, *TAR* = 58.03%, *DO* = 1 254, *PT* = 62, *NP* = 400 251) and 4 (*TN* = 6 499 928, *TAR* = 58.66%, *DO* = 1 258, *PT* = 64, *NP* = 403 077).

### Effectiveness of vaccination on the disease severity

**Fig. 23:**
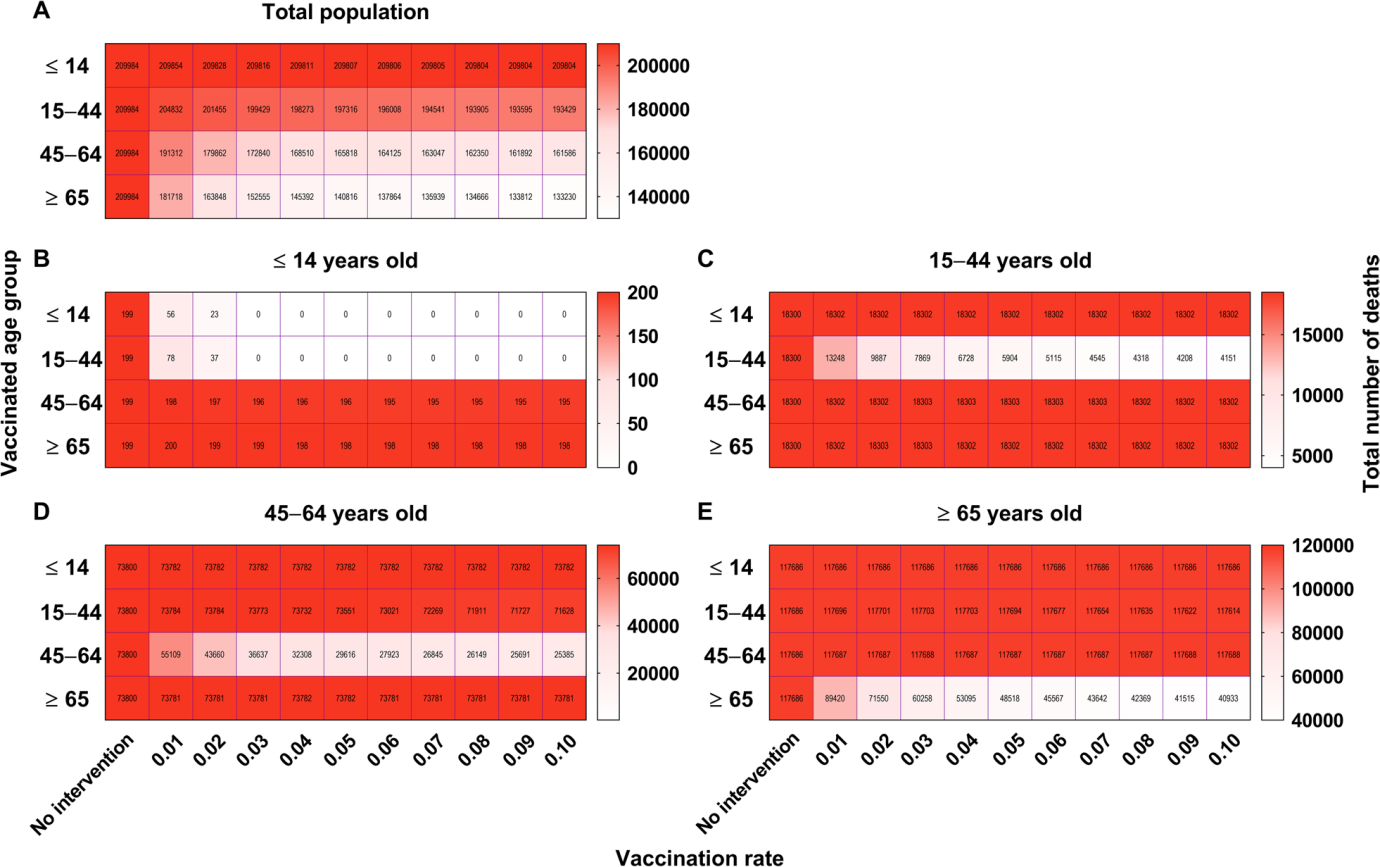
The influence from vaccination to severity in scene XIV to XVII of scenario II. **A** The number of deaths in each age group when vaccinated the total population. **B** The number of deaths in each age group, when vaccinated, ≤ 14 years old. **C** The number of deaths in each age group when vaccinated 15‒44 years old. **D** The number of deaths in each age group when vaccinated 45‒64 years old. **E** Number of deaths in each age group when vaccinated ≥ 65 years old

The simulated total number of deaths decreased with increasing age and vaccination rate (Fig. 23). The simulated total number of deaths was 209 984 in the total population, and it was 19 918 300, 73 800 and 117 686 in age groups 1, 2, 3, and 4, respectively. The best simulation for the total population was obtained with the vaccination of age group 4 (*δ* = 0.1, *ND* = 133 230), followed by vaccinating age group 3 (*δ* = 0.1, *ND* = 161 586). Moreover, the best simulation for decreasing the death rate of age group 1 was vaccination of group 1 (*δ* = 0.03, *ND* = 0), when the members of age group 2 were vaccinated (*δ* = 0.1, *ND* = 4 151), when the members of age group 3 were vaccinated (*δ* = 0.1, *ND* = 25 385), and when the members of age group 4 were vaccinated (*δ* = 0.1, *ND* = 40 933). Our findings suggest that vaccination protocols should prioritize older populations.

## Discussion

When modeling the transmissibility, although the transmission pattern varied from one area to another, the highest transmissibility was found in individuals aged 15–44 years, while the highest risk of infection was among the elderly population. Therefore, the optimal vaccination strategy for controlling the transmission of COVID-19 should be to first vaccinate about 90% of 15–44 years old, while for reducing the disease severity, the vaccination priority should be on the older population.

Similar to our previous study, the age-specific SEIAR model fits the data well [11, 12]. There is no doubt that it has a great impact on controlling the epidemics in Wuhan City [2], while accounting for key intervention measures such as travel ban, case isolation, and increasing social distancing, among others.

Before the travel ban of Wuhan City, the highest transmissibility was found within the most socially active age group, those aged 15–44 years old. The highest contact frequency in this group was directly linked to its high level of contact with other age groups [51]. January 25, 2020, corresponding to the Spring Festival (Chinese Lunar New Year celebration), an event where there would be a large number of travelers, such as workers and students returning home, present within the first 15 days of the festival [56, 57]. In China, the secondary cases were mostly caused by travelers, especially middle-aged and elderly people [31, 58]. Meanwhile, our study indicated that the susceptibility of those 15–65 years of age was higher than those in other age groups. If we set the period before the travel ban in Wuhan City as a baseline, the highest level of interaction was among those 15–65 years of age. Following the implementation of control measures during the travel ban period, transmissibility decreased in Wuhan City. The *R*_S_ value for the population below 14 years of age was 0.0255, and it gradually increased in stages 2 (*R*_S_ = 0.0386) and 3 (*R*_S_ = 0.0641). This finding suggests that the risk of transmission would be amplified after intervention for those below 14 years of age, especially during the travel ban and home quarantine. A previous study indicated that children and adults face a similar risk of infection [58]. Meanwhile, some surveys also reported that the transmission of SARS-CoV-2 largely occurred within the family [60, 61]. Therefore, it is important to prevent transmission within families after home isolation.

Although the contact frequency among ≥ 65 years old is very low (*α* = 1.91), this age class displayed the highest probability of infection upon single contact, that is, it is the most susceptible age class, (*q* = 15.144%) before the travel ban. In China, there are many cluster activities among the elderly, such as square dancing, dinner parties, and card games. These cluster activities have promoted long-term and effective exposure between people, thereby increasing the risk of infection [61]. Meanwhile, in the other three stages, the elderly are also at a very high risk of being infected by other age groups. This suggests that social distancing should be increased and cluster activities should be decreased to control COVID-19, especially in the elderly.

However, because of the varied results with influenza incidence in various age groups [62, 63], the transmission interaction in the age group was not a constant pattern. There is a major difference in age-specific transmission between Wuhan City, Hunan Province, and Jilin Province. This difference might be related to differing societal factors such as economy, culture, and demography. The population density and economic status of Wuhan City was higher than those of Hunan Province and Jilin Province. Some studies have indicated that there are differences in COVID-19 outbreaks in every province of China [58]. In addition, the epidemics of Hunan Province and Jilin Province were local infections mostly caused by imported cases. Therefore, age-specific transmission patterns need to be further explored and compared between different areas.

Currently, the mRNA vaccine of BioNTech and Moderna reported great effect (VE > 90%) for controlling the ancestral virus [5–7]. However, the effectiveness of the vaccine has been gradually decreasing with the mutation of the virus, exemplified by just 75% (95% *CI*: 68%–79%) of ChAdOx1 nCoV-19 for the Delta variant [8]. In this study, we considered VE-like vaccine efficacy of measles (VE = 90%), influenza B (VE = 70%) and influenza A (VE = 30%). We obtained similar age-optimized vaccination results for the above three conditions. Our findings suggest that the optimal vaccination proposal is consistent across age groups regardless of vaccine efficacy. VE is at least 70% and 90%, respectively, which is consistent with the actual vaccine effects. Therefore, the evaluation of COVID-19 vaccines could be referred to as the vaccine against influenza and measles. This study explored two scenarios including 19 scenes to explore the unknown future vaccine efficacy and behavior and the optimal strategy to control COVID-19. The simple model in the scenario I aim to cover a wide range of VE, and the more complex model in scenario II aims to explore the decreasing relative infectivity and susceptibility. Indeed, the vaccine for measles has a good efficacy [44], but this might not be the case for COVID-19 vaccines. Therefore, when simulating the efficacy of COVID-19 as that of the seasonal influenza vaccine, the vaccination coverage should be at least 87.93% of the total population in scene XI of scenario I and 96.53% of the total population in scene XIX of scenario II. Conversely, a specific study indicated that a vaccine with an efficacy ≥ 50% would be enough to mitigate the pandemic and the vaccine should be allocated for use in the elderly first [13, 64].

To increase the accuracy of the simulation, we further added asymptomatic infection as a factor and simulated the vaccination outcomes after estimation of transmissibility in different age groups according to the real-life situation.

Considering that it is difficult to vaccinate nearly 90% of the total population, we simulated the vaccination rate in each age group. Although several studies, including ours, indicated a high risk of infection in the elderly [12, 52], our results of vaccination modeling in each age group showed that the optimal strategy was to first vaccinate individuals aged 15–44 years. Whether this simulation corresponds to scenario I or scenario II, it can significantly reduce the number of cases in the overall population. The main reason is the very high transmissibility (including susceptibility and infectivity) of SARS-CoV-2 in individuals of the 15–44 age group. Furthermore, the age class with the highest contact frequency were those 15–44 years old [52]. The higher-risk occupations included car, taxi, and van drivers, shop sales associates, domestic housekeepers, religious professionals, etc. [65], who belonged mainly in the 15–44-year-old group. Therefore, a very positive outcome in terms of transmission can be expected when vaccinating first this age group, especially high-risk workers such as healthcare workers, drivers, transport workers, and services and sales workers. If the objective is to reduce mortality, the strategy should be to first target the age group above 65 [13, 29, 66]. In contrast, our results showed that we should not limit the vaccination to a given class but instead, to optimize the efficacy, vaccinate in a specific order: first those ≥ 65 years old, followed by those 45–64 years old, then those 15–44 years old, and finally those ≤ 14 years old.

However, transmission patterns differ from one area to another [67, 68]. We highlight this heterogeneity in transmission, especially in terms of age interaction, because these results may be optimal for Wuhan City but perhaps less suitable for other regions. Therefore, we should estimate a strategy of vaccination optimization after having sufficiently clarified age-specific transmission interactions in different areas. In particular, we should estimate the transmissibility and simulate vaccination outcomes in different age groups in different regions. We limited the real-time vaccinating process simulated in our model, *δ* = 0.1 means nearly 10% of the total population is vaccinated per day. It is necessary to simulate an initial proportion of the immune population. A study reported the impact of policy interventions (like home quarantine) and meteorological factors (such as air index, temperature, precipitation, and relative humidity) on vaccination effectiveness [69]. Although an immune barrier has been established in a proportion of people, we need to consider the impact of meteorological factors on transmission and vaccination and simulate the combined effect between vaccination and non-pharmacological interventions in future work.

### Limitations

In the study, we should collect most COVID-19 data to compare the different age-specific transmission in various areas. Furthermore, we are limited to not analyzing the impact of meteorological factors on transmission and vaccination. At last, we should re-evaluate the optimization strategy based on the current immune barrier.

## Conclusions

The highest transmissibility was observed in those aged 15–44 years and the risk of infection probability was highest in the elderly. In China, approximately 85% of the total population should be vaccinated to effectively build an immune barrier and take reopening under consideration. The optimized strategy to control transmission was to first vaccinate about 90% of individuals aged 15–44 years, but for reducing the disease severity, the elderly should be vaccinated first.

## Supplementary Information

**Additional file 1: Table S1.** The data of illness onset of four age groups in Wuhan City.

**Additional file 2: Text S1.** Equations of Model 2 and Model 3.

**Additional file 3: Table S2.** Description and source of parameters in the age-specific model.

**Additional file 4: Table S3.** Five indicators and vaccination coverage of scene XI and scene XIX.

**Additional file 5: Tables S4‒S7.** Simulation different vaccination scenes.

**Additional file 6: Tables S8‒S11.** Simulated different vaccination rates.

## Data Availability

All relevant data are within the paper and its Additional Information.

## Abbreviations

COVID-19: Coronavirus disease 2019
NPIs: Non-pharmaceutical interventions
VE: Vaccine efficacy
SARS-CoV-2: Severe acute respiratory syndrome coronavirus 2
SEIR: Susceptible-exposed-infectious-removed
SEIAR: Susceptible–exposed–symptomatic–asymptomatic–recovered/removed
*R*_eff_: Effective reproduction number
*R*_I_: Infectivity
*R*_S_: Susceptibility
*q*: Probability of infection from a single contact
*NT*: Total number of new cases
*TAR*: Total attack rate
*NP*: Number of new cases at peak
*DO*: Duration of outbreak
*PT*: Peak time
*ND*: Total number of deaths
*R*^2^: Coefficient of determination
