# Supplementary material for: The optimal vaccination strategy to control COVID-19: a modeling study in Wuhan City, China
Source: Infect Dis Poverty. 2021 Dec 28;10:140. doi: 10.1186/s40249-021-00922-4 (PMC8712277; doi:10.1186/s40249-021-00922-4)
Supplement: Supplementary file 6 — Additional file 6: Tables S8‒S11. Simulated different vaccination rates. [file 40249_2021_922_MOESM6_ESM.docx]

**Text S1. Equations of Model 2 and Model 3.**

The equations used in the model (Model 2) are as follows:

$$i\neq j$$

$$\frac{dS_{i}}{dt}=-\beta_{ii}S_{i}\left( I_{i}+\kappa A_{i} \right)-\beta_{ji}S_{i}\left( I_{j}+\kappa A_{j} \right)-\delta_{i}S_{i}$$

$$\frac{dE_{i}}{dt}=\beta_{ii}{(V}_{1i}+({1-\lambda)V_{2i}+S}_{i})\left( I_{i}+\kappa A_{i} \right)+\beta_{ji}{(V}_{1i}+({1-\lambda)V_{2i}+S}_{i})\left( I_{j}+\kappa A_{j} \right)-\left( 1-p \right)\omega E_{i}-p\omega^{'}E_{i}$$

$$\frac{dI_{i}}{dt}=\left( 1-p \right)\omega E_{i}-\gamma I_{i}-f_{i}I_{i}$$

$$\frac{dA_{i}}{dt}=p\omega^{'}E_{i}-\gamma^{'}A_{i}$$

$$\frac{dR_{i}}{dt}={\gamma I}_{i}+{\gamma^{'}A}_{i}$$

$$\frac{dV_{1i}}{dt}=\delta_{i}S_{i}-\beta_{ii}V_{1i}\left( I_{i}+\kappa A_{i} \right)-\beta_{ji}V_{1i}\left( I_{j}+\kappa A_{j} \right)-V_{1i}$$

$$\frac{dV_{2i}}{dt}=V_{1i}-{\left( 1-\lambda\right)\beta}_{ii}V_{2i}\left( I_{i}+\kappa A_{i} \right)-{(1-\lambda)\beta}_{ji}V_{2i}\left( I_{j}+\kappa A_{j} \right)$$

$$\frac{dS_{j}}{dt}=-\beta_{jj}S_{j}\left( I_{j}+\kappa A_{j} \right)-\beta_{ji}S_{j}\left( I_{i}+\kappa A_{i} \right)-\delta S_{j}$$

$$\frac{dE_{j}}{dt}=\beta_{jj}{(V}_{1j}+({1-\lambda)V_{2j}+S}_{j})\left( I_{j}+\kappa A_{j} \right)+\beta_{ji}{(V}_{1j}+({1-\lambda)V_{2j}+S}_{j})\left( I_{i}+\kappa A_{i} \right)-\left( 1-p \right)\omega E_{j}-p\omega^{'}E_{j}$$

$$\frac{dI_{j}}{dt}=\left( 1-p \right)\omega E_{j}-\gamma I_{j}-f_{i}I_{j}$$

$$\frac{dA_{j}}{dt}=p\omega^{'}E_{j}-\gamma^{'}A_{j}$$

$$\frac{dR_{j}}{dt}={\gamma I}_{j}+{\gamma^{'}A}_{j}$$

$$\frac{dV_{1j}}{dt}=\delta_{j}S_{j}-\beta_{jj}V_{1j}\left( I_{j}+\kappa A_{j} \right)-\beta_{ij}V_{1j}\left( I_{i}+\kappa A_{i} \right)-V_{1j}$$

$$\frac{dV_{2j}}{dt}=V_{1j}-{\left( 1-\lambda\right)\beta}_{jj}V_{2j}\left( I_{j}+\kappa A_{j} \right)-{(1-\lambda)\beta_{ij}V}_{2j}\left( I_{i}+\kappa A_{i} \right)$$

$$N=S_{i}+E_{i}+I_{i}+A_{i}+R_{i}+V_{1i}+V_{2i}$$

The equations used in the model (Model 3) are as follows:

$$i\neq j$$

$$\frac{dS_{i}}{dt}=-\beta_{ii}S_{i}{((I}_{1i}+\kappa A_{1i})+{y(I}_{2i}+\kappa A_{2i}))-\beta_{ji}S_{i}{((I}_{1j}+\kappa A_{1j})+{y(I}_{2j}+\kappa A_{2j}))-\delta_{i}S_{i}-\beta_{ii}\theta_{i}S_{i}(x(I_{1i}+\kappa A_{1i})+z(I_{2i}+\kappa A_{2i})){-\beta}_{ji}\theta_{i}S_{i}(x(I_{1j}+\kappa A_{1j})+z(I_{2j}+\kappa A_{2j}))$$

$$\frac{dE_{1i}}{dt}=\beta_{ii}S_{i}{((I}_{1i}+\kappa A_{1i})+{y(I}_{2i}+\kappa A_{2i}))+\beta_{ji}S_{i}{((I}_{1j}+\kappa A_{1j})+{y(I}_{2j}+\kappa A_{2j}))+\beta_{ii}V_{1i}{((I}_{1i}+\kappa A_{1i})+{y(I}_{2i}+\kappa A_{2i}))+\beta_{ji}V_{1i}{((I}_{1j}+\kappa A_{1j})+{y(I}_{2j}+\kappa A_{2j}))-\left( 1-p \right)\omega E_{1i}-p\omega^{'}E_{1i}$$

$$\frac{dI_{1i}}{dt}=\left( 1-p \right)\omega E_{1i}-\gamma I_{1i}-f_{i}I_{1i}$$

$$\frac{dA_{1i}}{dt}=p\omega^{'}E_{1i}-\gamma^{'}A_{1i}$$

$$\frac{dR_{1i}}{dt}={\gamma I}_{1i}+{\gamma^{'}A}_{1i}$$

$$\frac{dV_{1i}}{dt}=\delta_{i}S_{i}-\beta_{ii}S_{1i}{((I}_{1i}+\kappa A_{1i})+{y(I}_{2i}+\kappa A_{2i}))-\beta_{ji}V_{1i}{((I}_{1j}+\kappa A_{1j})-{y(I}_{2j}+\kappa A_{2j})- V_{1i}$$

$$\frac{dV_{2i}}{dt}=V_{1i}-\beta_{ii}V_{2i}(x\left( I_{1i}+\kappa A_{1i} \right)+z(I_{2i}+\kappa A_{2i})){-\beta}_{ji}V_{2i}(x(I_{1j}+\kappa A_{1j})+z(I_{2j}+\kappa A_{2j}))$$

$$\frac{dE_{2i}}{dt}=\beta_{ii}\theta_{i}S_{i}\left( x\left( I_{1i}+\kappa A_{1i} \right)+z\left( I_{2i}+\kappa A_{2i} \right) \right){+\beta}_{ji}\theta_{i}S_{i}\left( x\left( I_{1j}+\kappa A_{1j} \right)+z\left( I_{2j}+\kappa A_{2j} \right) \right)+\beta_{ii}V_{2i}(x\left( I_{1i}+\kappa A_{1i} \right)+z(I_{2i}+\kappa A_{2i})){+\beta}_{ji}V_{2i}(x(I_{1j}+\kappa A_{1j})+z(I_{2j}+\kappa A_{2j})) -\left( 1-p \right)\omega E_{2i}-p\omega^{'}E_{2i}$$

$$\frac{dI_{2i}}{dt}=\left( 1-p \right)\omega E_{2i}-\gamma I_{2i}-zf_{i}I_{2i}$$

$$\frac{dA_{2i}}{dt}=p\omega^{'}E_{2i}-\gamma^{'}A_{2i}$$

$$\frac{dR_{2i}}{dt}={\gamma I}_{2i}+{\gamma^{'}A}_{2i}$$

$$\frac{dS_{j}}{dt}=-\beta_{jj}S_{j}{((I}_{1j}+\kappa A_{1j})+{y(I}_{2j}+\kappa A_{2j}))-\beta_{ij}S_{j}{((I}_{1i}+\kappa A_{1i})+{y(I}_{2i}+\kappa A_{2i}))-\delta_{j}S_{j}-\beta_{jj}\theta_{j}S_{j}(x(I_{1j}+\kappa A_{1j})+z(I_{2j}+\kappa A_{2j})){-\beta}_{ij}\theta_{j}S_{j}(x(I_{1i}+\kappa A_{1i})+z(I_{2i}+\kappa A_{2i}))$$

$$\frac{dE_{1j}}{dt}=\beta_{jj}S_{j}{((I}_{1j}+\kappa A_{1j})+{y(I}_{2j}+\kappa A_{2j}))+\beta_{ij}S_{j}{((I}_{1i}+\kappa A_{1i})+{y(I}_{2i}+\kappa A_{2i}))+\beta_{jj}V_{1j}{((I}_{1j}+\kappa A_{1j})+{y(I}_{2j}+\kappa A_{2j}))+\beta_{ij}V_{1j}{((I}_{1i}+\kappa A_{1i})+{y(I}_{2i}+\kappa A_{2i}))-\left( 1-p \right)\omega E_{1j}-p\omega^{'}E_{1j}$$

$$\frac{dI_{1j}}{dt}=\left( 1-p \right)\omega E_{1j}-\gamma I_{1j}-f_{j}I_{1j}$$

$$\frac{dA_{1j}}{dt}=p\omega^{'}E_{1j}-\gamma^{'}A_{1j}$$

$$\frac{dR_{1j}}{dt}={\gamma I}_{1j}+{\gamma^{'}A}_{1j}$$

$$\frac{dV_{1j}}{dt}=\delta_{j}S_{j}-\beta_{jj}V_{1j}{((I}_{1j}+\kappa A_{1j})+{y(I}_{2j}+\kappa A_{2j}))-\beta_{ij}V_{1j}{((I}_{1i}+\kappa A_{1i})+{y(I}_{2i}+\kappa A_{2i}))- V_{1j}$$

$$\frac{dV_{2j}}{dt}=V_{1j}-\beta_{jj}V_{2j}(x(I_{1j}+\kappa A_{1j})+z(I_{2j}+\kappa A_{2j})){-\beta}_{ij}V_{2ji}(x(I_{1i}+\kappa A_{1i}+z(I_{2i}+\kappa A_{2i}))$$

$$\frac{dE_{2j}}{dt}=\beta_{jj}V_{2j}(x(I_{1j}+\kappa A_{1j})+z(I_{2j}+\kappa A_{2j})){+\beta}_{ij}V_{2j}(x\left( I_{1i}+\kappa A_{1i})+z\left( I_{2i}+\kappa A_{2i} \right) \right)+\beta_{jj}\theta_{j}S_{j}(x(I_{1j}+\kappa A_{1j})+z(I_{2j}+\kappa A_{2j})){+\beta}_{ij}\theta_{j}S_{j}(x(I_{1i}+\kappa A_{1i})+z(I_{2i}+\kappa A_{2i})) -\left( 1-p \right)\omega E_{2j}-p\omega^{'}E_{2j}$$

$$\frac{dI_{1j}}{dt}=\left( 1-p \right)\omega E_{2j}-\gamma I_{2j}-{zf}_{j}I_{2j}$$

$$\frac{dA_{2j}}{dt}=p\omega^{'}E_{2j}-\gamma^{'}A_{2j}$$

$$\frac{dR_{2j}}{dt}={\gamma I}_{2j}+{\gamma^{'}A}_{2j}$$

$$N=S_{i}+E_{1i}+I_{1i}+A_{1i}+R_{1i}+V_{1i}+V_{2i}{+ E}_{2i}+I_{2i}+A_{2i}+R_{2i}$$
